# Time-Resolved Expression Profiling of the Nuclear Receptor Superfamily in Human Adipogenesis

**DOI:** 10.1371/journal.pone.0012991

**Published:** 2010-09-27

**Authors:** Mari Lahnalampi, Merja Heinäniemi, Lasse Sinkkonen, Martin Wabitsch, Carsten Carlberg

**Affiliations:** 1 Department of Biosciences, University of Eastern Finland, Kuopio, Finland; 2 Life Sciences Research Unit, University of Luxembourg, Luxembourg, Luxembourg; 3 Division of Pediatric Endocrinology and Diabetes, University of Ulm, Ulm, Germany; Ecole Normale Supérieure de Lyon, France

## Abstract

**Background:**

The differentiation of fibroblast-like pre-adipocytes to lipid-loaded adipocytes is regulated by a network of transcription factors, the most prominent one being the nuclear receptor peroxisome proliferator-activated receptor (PPAR) γ. However, many of the other 47 members of the nuclear receptor superfamily have an impact on adipogenesis, which in human cells has not been investigated in detail.

**Methodology/Principal Findings:**

We analyzed by quantitative PCR all human nuclear receptors at multiple time points during differentiation of SGBS pre-adipocytes. The earliest effect was the down-regulation of the genes *RARG*, *PPARD*, *REV-ERBA*, *REV-ERBB*, *VDR* and *GR* followed by the up-regulation of *PPARG*, *LXRA* and *AR.* These observations are supported with data from 3T3-L1 mouse pre-adipocytes and primary human adipocytes. Investigation of the effects of the individual differentiation mix components in short-term treatments and of their omission from the full mix showed that the expression levels of the early-regulated nuclear receptor genes were most affected by the glucocorticoid receptor (GR) ligand cortisol and the phosophodiesterase inhibitor IBMX. Interestingly, the effects of both compounds converged to repress the genes *PPARD*, *REV-ERBA*, *REV-ERBB*, *VDR* and *GR*, whereas cortisol and IBMX showed antagonistic interaction for *PPARG*, *LXRA* and *AR* causing a time lag in their up-regulation. We hypothesize that the well-known auto-repression of GR fine-tunes the detected early responses. Consistently, chromatin immunoprecipitation experiments showed that GR association increased on the transcription start sites of the genes *RARG*, *REV-ERBB*, *VDR* and *GR*.

**Conclusions/Significance:**

Adipocyte differentiation is a process, in which many members of the nuclear receptor superfamily change their mRNA expression. The actions of cortisol and IBMX converged to repress several nuclear receptors early in differentiation, while up-regulation of other nuclear receptor genes showed a time lag due to antagonisms of the signals. Our results place GR and its ligand cortisol as central regulatory factors controlling early regulatory events in human adipogenesis that precedes the regulation of the later events by *PPARG*.

## Introduction

Adipose tissue is an important metabolic organ and has a central role in energy balance and glucose homeostasis [1]. Due to the worldwide epidemic of obesity there is a need for a deeper understanding of the mechanisms controlling the development of adipocytes [2]. The differentiation of fibroblast-like pre-adipocytes to lipid-loaded adipocytes is regulated by a network of transcription factors, the most prominent one being the nuclear receptor peroxisome proliferator-activated receptor (PPAR) γ [3]. During the differentiation of 3T3-L1 mouse pre-adipocytes the number of genomic binding sites for PPARγ increases from a few to more than 5,000 [4]; [5], which implicates that this nuclear receptor regulates hundreds of genes during adipogenesis.

Nuclear receptors form a transcription factor family with 48 human members, most of which have the special property to be ligand-activated [6]; [7]. This property has attracted interest in nuclear receptor family members as possible therapeutic targets. For example, the synthetic PPARγ ligand rosiglitazone is used in the therapy of type 2 diabetes mainly acting via its effects on gene regulation in adipocytes [8]. Nuclear receptors belong to the best-characterized representatives of approximately 3,000 different mammalian proteins that are involved in transcriptional regulation in human tissues [9]. They modulate genes that affect processes as diverse as reproduction, development, inflammation and general metabolism. Nuclear receptors can be classified based on ligand sensitivity [6], evolution of nuclear receptor genes [10] and their physiological role as interpreted from tissue-specific expression patterns [11].

The ligand sensitivity approach suggests three nuclear receptor classes [6]. Class I contains the endocrine receptors with high-affinity hormonal lipids, such as the receptors for the steroid hormones estradiol, progesterone, testosterone, cortisol and aldosterol, for triiodothyronine (T_3_) and for the biologically active forms of the fat-soluble vitamins A and D, all-*trans* retinoic acid and 1α,25-dihydroxyvitamin D_3_. These 12 nuclear receptors can be defined functionally as being able to bind their specific ligand with a K_d_ of 1 nM or less [6]. In class II are adopted orphan receptors that bind to dietary lipids and xenobiotics in the micro- to millimolar concentration range [12]; [13], such as PPARs α, δ and γ, liver X receptors (LXRs) α and β, retinoid X receptors (RXRs) α, β and γ, farnesoid X receptor, constitutive androstane receptor, pregnane X receptor, retinoid orphan receptors (ROR) α and β and reverse-ErbA (Rev-ErbA) α and β. Finally, in class III are orphan receptors, such as liver receptor homolog 1 (LRH-1), which seem not to posses a physiological ligand [14]; [15].

SGBS human pre-adipocytes derive from the stromal cell fraction of subcutaneous adipose tissue of an infant with Simpson-Golabi-Behmel syndrome [16] and have been shown to represent a good model to study adipocyte function. Therefore, these cells are an interesting source of human pre-adipocytes with high capacity for adipose differentiation [17]. The cells are induced to differentiate with the thyroid hormone receptor (TR) ligand T_3_, the GR ligand cortisol, insulin, the PPARγ ligand rosiglitazone and the phosphodiesterase inhibitor isobutylmethylxanthine (IBMX). The cells take some 10–12 days until terminal differentiation as monitored by the production of lipid droplets. The widely used 3T3-L1 mouse pre-adipocytes [18] differentiate within 6 days upon exposure to a very similar differentiation mix, although typically T_3_ is omitted and fetal bovine serum (FBS) is included in the medium.

Since both nuclear receptor ligands as well as nuclear receptor gene expression play a central role in adipogenesis, we took in this study the approach to profile the time-resolved expression of all nuclear receptor superfamily members in the conversion of human pre-adipocytes to adipocytes. Emphasis is given to those nuclear receptor genes that are regulated in early phases of adipogenesis, such as *PPARG*, *AR*, *RARG*, *PPARD*, *REV-ERBA*, *REV-ERBB*, *VDR* and *GR*. These observations are supported by data from 3T3-L1 cells and primary human adipocytes and comparison to published and our own novel data from mouse 3T3-L1 cells suggest a high level of conservation between mouse and human adipogenesis. From the individual differentiation mix components we demonstrated cortisol and IBMX to have the strongest effect on the expression of early-regulated nuclear receptors. Interestingly, the signaling pathways triggered by these compounds converged to repress several nuclear receptors, but an antagonistic interaction was seen for up-regulated genes, causing a time lag in their induction profile. Chromatin immunoprecipitation (ChIP) experiments show an increase of GR association with the transcription start sites (TSSs) of *RARG*, *REV-ERBB*, *VDR* and *GR*, which are early down-regulated by cortisol treatment. The auto-repression by cortisol fine-tunes the expression of *GR* and may speed up the response during the early steps of adipogenesis and later control the steady-state levels reached.

## Results

### Nuclear receptor expression profiling during SGBS and 3T3-L1 differentiation

**Figure 1:**
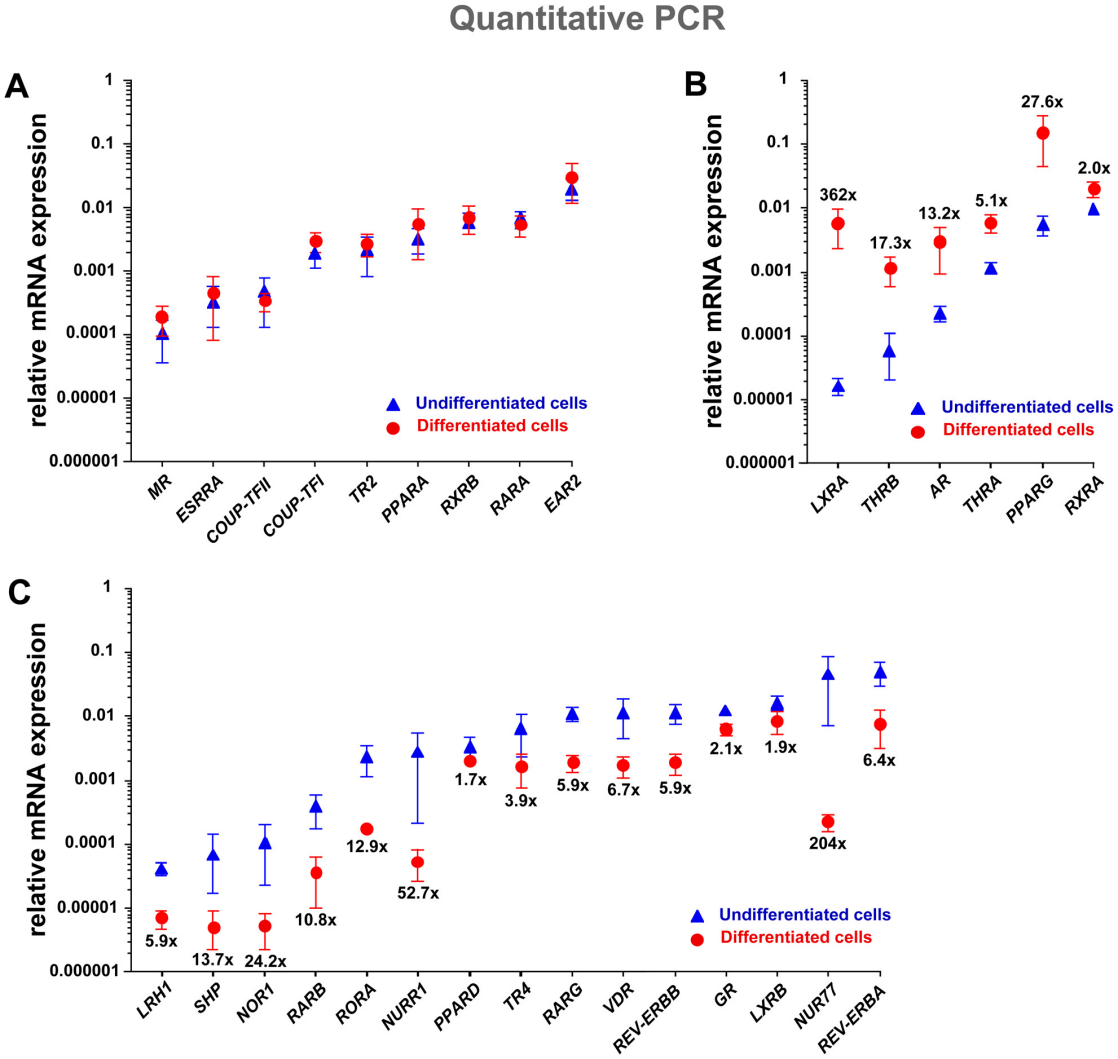
Nuclear receptor mRNA expression in undifferentiated and differentiated human SGBS cells. Real-time quantitative PCR with gene-specific primers was used to determine the mRNA expression levels of all 48 nuclear receptor genes in relation to the housekeeping gene *RPL13A* in undifferentiated (blue triangle) or 12 days differentiated (red circle) SGBS cells. Thirty nuclear receptor genes were found to be expressed, of which due to differentiation nine did not change their expression (A), six were up-regulated (B) and 15 were down-regulated (C). Fold changes were calculated in reference to undifferentiated cells. Data points represent the means of three biological repeats and the bars indicate standard deviations.

The early transcriptional cascades activated at the initiation of cell differentiation determine alternate cell fates and their commitment to terminal differentiation. In order to identify which members of the nuclear receptor superfamily are expressed during human adipocyte differentiation, we determined by real-time quantitative PCR the relative mRNA expression profile of all 48 nuclear receptor genes in undifferentiated and 12 days differentiated human SGBS cells (Fig. 1). We detected 30 nuclear receptor genes being expressed and only nine of them did not change their expression level during the differentiation process (Fig. 1A). From the remaining 21 nuclear receptor genes, six (*THRA*, *THRB*, *PPARG*, *LXRA*, *RXRA* and *AR*) were between 2- and 362-fold up-regulated (Fig. 1B), while 15 were between 1.7- and 204-fold down-regulated (Fig. 1C). In undifferentiated pre-adipocytes the *REV-ERBA* gene was highest expressed, while the most prominent nuclear receptor gene in differentiated SGBS cells was the 27.6-fold up-regulated *PPARG* gene. The latter observation is in good accordance with the literature [4]; [5]. The most up-regulated gene was *LXRA*, which was very low expressed in pre-adipocytes, but increased 362-fold during differentiation, an observation that has been previously described also in mouse cells [19]. In contrast, the most down-regulated nuclear receptor gene was *NUR77*, which was second ranking in undifferentiated cells, but lost its expression by a factor of 204. Interestingly, also the two other members of this nuclear receptor sub-family, *NURR1* and *NOR1*, were significantly down-regulated (52.7- and 24.2-fold). In contrast, in mouse adipogenesis the expression of the NR4A subfamily members was reported to be up-regulated [20]–[22].

**Figure 2:**
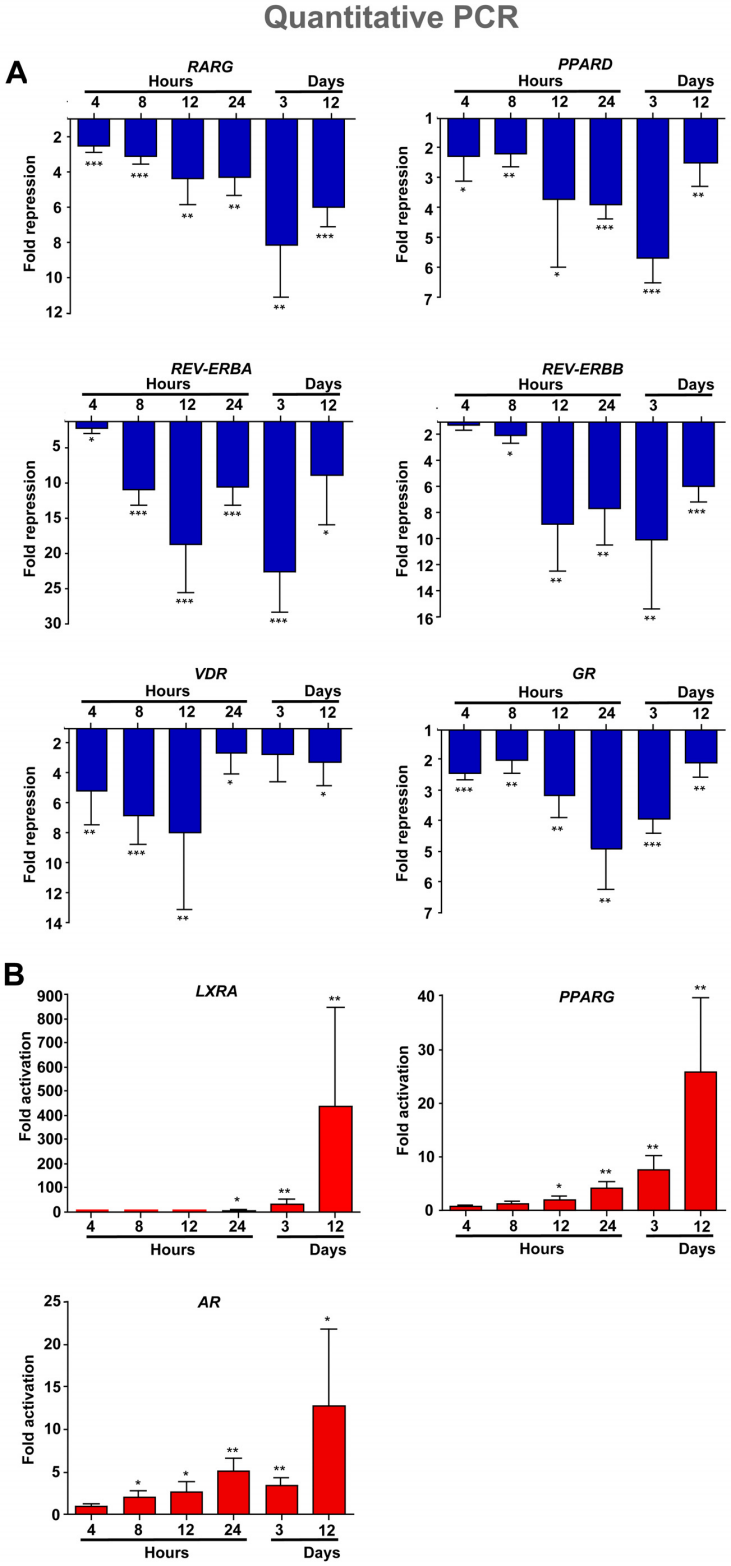
Time profile of nuclear receptor expression during human SGBS pre-adipocyte differentiation. Real-time quantitative PCR was performed in order to determine mRNA expression of nuclear receptor genes in relation to the housekeeping gene *RPL13A* at indicated time points of SGBS differentiation. Six early repressed genes are shown (A) and the first three up-regulated genes (B). Columns represent the means of at least three biological repeats and the bars indicate standard deviations. Two-tailed paired Student's t-tests were performed to determine the significance of the mRNA level changes in reference to undifferentiated pre-adipocytes (* p<0.05, ** p<0.01, *** p<0.001).

For a more detailed investigation of the timing of the changes in the expression of nuclear receptors during the 12 days of SGBS differentiation, we determined by real-time quantitative PCR the relative mRNA expression of the 30 nuclear receptor genes that showed significant expression at time points 4 h, 8 h, 12 h, 24 h, 3 d and 12 d (Figs. 2 and S1). We could identify four groups with a consistent change in expression level among the nuclear receptor profiles. The earliest effect observed was the down-regulation of the six NR genes *RARG*, *PPARD*, *REV-ERBA*, *REV-ERBB*, *VDR* and *GR* (Fig. 2A). These genes responded already 4 to 8 h after initiation of differentiation and were maximally affected between time points 12 h and 3 d. This wave of repression was followed and later accompanied by the up-regulation of the genes *AR, PPARG* and *LXRA*, taking place at 8 h, 12 h and 24 h, respectively (Fig. 2B). Interestingly, the up-regulated genes showed their maxima at the end of the differentiation process. The up-regulation of three other genes, *THRA*, *THRB* and *RXRA*, was observed, but the effect took several days to manifest (Fig. S1). Furthermore, the genes *RORA*, *LXRB*, *TR4*, *NUR77* and *SHP* are late down-regulated (Fig. S1). Finally, the genes *RARA*, *RARB*, *PPARA*, *ESRRB*, *TR2*, *COUP-TFI*, *COUP-TFII*, *EAR2*, *ESRRA*, *MR*, *NURR1*, *NOR1* and *LRH1* show a mixed profile of up- and down-regulation (Fig. S1) and most of them do not significantly change their expression level, when comparing pre-adipocytes and differentiated adipocytes (Fig. 1A).

To compare the early regulation profile of human SGBS cells with that of mouse 3T3-L1 cells, we tested the expression of the mouse orthologues of the above six early repressed nuclear receptor genes and the first three up-regulated genes (Fig. S2). Eight of the nine genes were expressed, but we could not detect any expression of *Ar.* The detailed time course (4 h, 8 h, 12 h, 24 h, 3 d and 6 d) of the expression of the eight nuclear receptor genes in 3T3-L1 cells (Fig. S3) indicated that *Pparg* and *Lxra* are strongly up-regulated, *Rarg* is constantly down-regulated, while *Rev-erba*, *Rev-erbb* and *Gr* are down-regulated during the first one to three days (Fig. S3). This is in agreement with the data from the SGBS model. In contrast, *Ppard* is only modestly up-regulated at day 6 and *Vdr* is transiently up-regulated during the first 24 h (Fig. S3).

A comparison of nuclear receptor gene expression in public microarray data obtained from undifferentiated and 30 days differentiated primary human adipocytes [23] (Fig. S4) is in good accordance with our basal level data from the human SGBS cells. The group of genes that has a profound constant up-regulation during differentiation is observed to change also in primary human adipocytes: *LXRA* (338-fold), *PPARG* (85-fold), *RXRA* (2.3-fold), *AR* (2.3-fold) and *EAR2* (1.9-fold). The longer differentiation protocol used is expected to miss the early events that we observed, indeed the late repressed *RORA* was the only gene found to be down-regulated (2.2-fold).

Taken together, the adipocyte differentiation process is shown to result in major changes in the nuclear receptor transcriptional network. The profound repression of multiple nuclear receptor genes characterized the initiation of differentiation and a time lag was observed for all up-regulated nuclear receptors.

### Effect of differentiation mix compounds on early regulated nuclear receptor genes in SGBS and 3T3-L1 cells

To characterize the signaling pathways that lead to the initiation of the early regulatory cascades observed, we tested what effect the individual components of the adipocyte differentiation mix (T_3_, cortisol, insulin, rosiglitazone and IBMX) have on the expression of the six early down-regulated nuclear receptor genes in human SGBS cells and in mouse 3T3-L1 cells (Fig. S5). The three nuclear receptors that were up-regulated during the first day, albeit with a time lag, provide a comparison. The different compounds were tested first when used alone for a short-term treatment (4 h) and second when 24 h stimulation was performed with the full mix lacking one component. The addition or omission of cortisol affected the expression of the human genes *RARG*, *PPARD*, *REV-ERBA*, *VDR* and *GR* and *AR*, while in mice the genes *Pparg*, *Rev-erba*, *Rev-erbb*, *Vdr* and *Gr were affected*. Interestingly, IBMX affected the expression of the human genes *RARG*, *PPARD*, *PPARG*, *LXRA*, *VDR* and *GR. In mice IBMX regulated the* genes *Pparg*, *Rev-erba*, *Rev-erbb*, *Lxra*, *Vdr* and *Gr*. Only very modest effects were seen with the other compounds. Rosiglitazone affected only the level of *Pparg*, *Rev-erba*, *LXRA* and *Vdr* in mice, and LXRA in human, when omitted from the mix. T_3_ had an impact on the levels *Rev-erba* in mice and *Gr* in human. Insulin affected the levels of *VDR* in both species.

In summary, both in human SGBS cells as well as in mouse 3T3-L1 cells the differentiation mix components cortisol and IBMX had the broadest effects on early regulated nuclear receptor genes. We therefore focused further investigations on these two compounds.

### Effects of cortisol and IBMX on early regulated nuclear receptor genes

**Figure 3:**
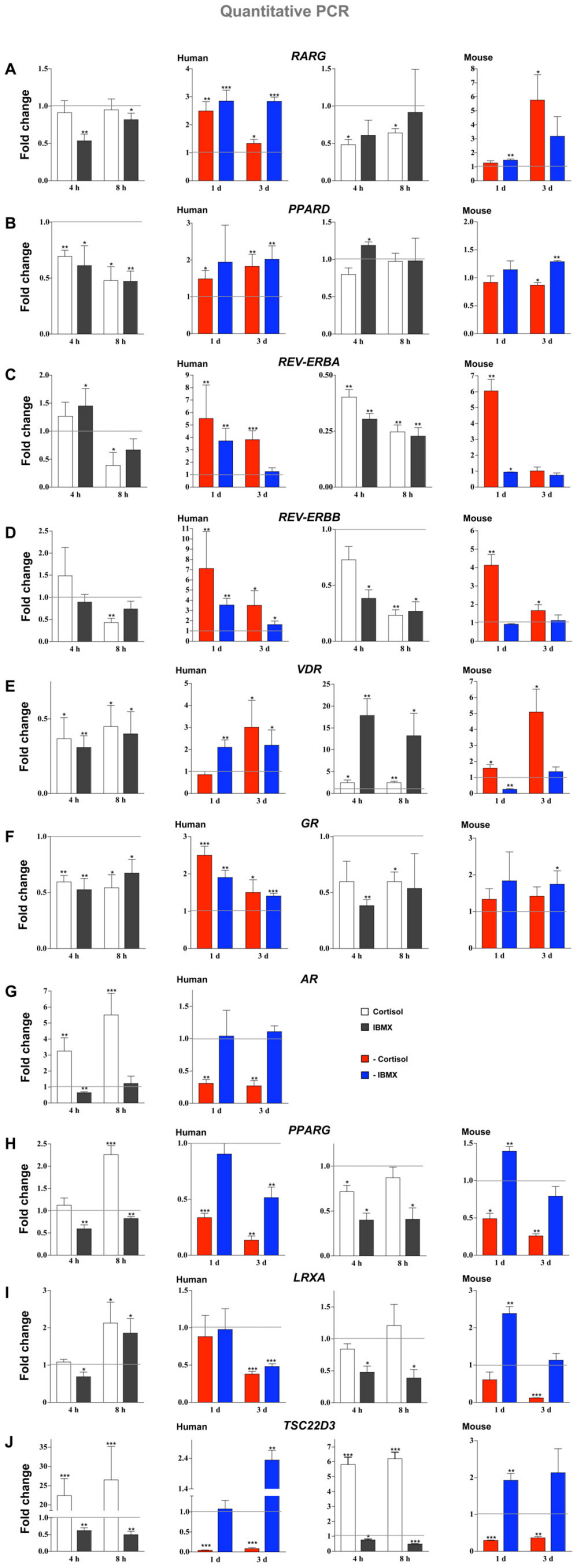
Effects of cortisol and IBMX on human SGBS and mouse 3T3-L1 differentiation. Real-time quantitative PCR was performed in order to determine mRNA expression of six repressed nuclear receptor genes (A–F), the comparison set of three up-regulated genes (G–I) and the reference gene *TSC22D3* (J) in relation to the housekeeping genes *RPL13A* and *Rplp0* in SGBS and 3T3-L1 cells, respectively. The cells were either stimulated for 4 and 8 h with cortisol or IBMX or they were differentiated for 1 or 3 d with full differentiation medium lacking the indicated compounds (either cortisol or IBMX). It should be noted that the *Ar* gene is not significantly expressed in 3T3-L1 cells. Columns represent the means of at least three biological repeats and the bars indicate standard deviations. Two-tailed paired Student's t-tests were performed to determine the significance of the mRNA level changes in reference to undifferentiated pre-adipocytes (for 4 and 8 h treatments) or in reference to full differentiation medium (for 1 and 3 d treatments) (* p<0.05, ** p<0.01, *** p<0.001).

For a more detailed study on the effects of cortisol and IBMX on the six repressed nuclear receptor genes (*RARG*, *PPARD*, *REV-ERBA*, *REV-ERBB*, *VDR* and *GR)*, and as comparison the three up-regulated genes (*AR*, *PPARG* and *LXRA*), we stimulated both human SGBS and mouse 3T3-L1 cells with the two compounds individually for 4 and 8 h or omitted them from the differentiation medium for one or three days (Fig. 3).

Among the down-regulated genes, coherent repressive effects of cortisol and IBMX were observed in both species. The human *RARG* gene was down-regulated after 4 h (1.9-fold) and 8 h (1.2-fold) by IBMX, but was not affected in this time frame by cortisol, although our previous experiment indicated that (Fig. 3A). However, the lack of both compounds for one and three days resulted in a 1.3- to 2.8-fold reduction in the repression of the gene. For the mouse orthologue *Rarg* early down-regulation after 4 h (2.1-fold) and 8 h (1.6-fold) by cortisol and the reduced repression (5.8-fold) after three days omitting cortisol from the medium could be observed. Although not fully consistent, these data support both cortisol and IBMX mediated repression of *RARG*. The gene *PPARD* was 1.4- to 2.1-fold repressed after early cortisol or IBMX treatment and IBMX and 1.5- to 2-fold higher expressed, when either of the two compounds was missing from the differentiation mix (Fig. 3B). In contrast, the mouse gene *Ppard* was slightly up-regulated (1.2-fold) after 4 h IBMX treatment and significantly affected by omission of the two compounds at day 3. Therefore, the repression in human cells can be concluded to be IBMX- and cortisol-mediated. The *REV-ERBA* gene was 2.6-fold repressed after 8 h cortisol treatment and 1.5-fold induced after 4 h IBMX stimulation (Fig. 3C). The omission of cortisol reduced the repression of the gene 5.5-fold after one day and 3.8-fold after three days, while one day lacking IBMX resulted in a 3.7-fold weaker repression, again supporting the conclusions made before that the repression effects seem to be mediated by mutualism of the cortisol and IBMX treatments. The repression by cortisol after 8 h and the induction of the one day cortisol omission could be confirmed for the *Rev-erba* gene. The *REV-ERBB* gene (Fig. 3D) was 2.3-fold repressed after 8 h cortisol treatment and the omission of cortisol reduced the repression of the gene 7.1-fold after one day and 3.5-fold after three days. The repression by cortisol after 8 h and the reduction in this repression by the one and three days cortisol omission could be confirmed for the mouse orthologue *Rev-erbb*. The mouse gene was also responsive to IBMX at the early time points, whereas these effects were only observed at days 1 and 3 in human cells. The *VDR* gene was 2.2- to 3.2-fold repressed after 4 and 8 h cortisol or IBMX stimulation, 3-fold higher after three days cortisol omission and 2.1- and 2.2-fold higher after one and three days culturing without IBMX as compared to full differentiation mix (Fig. 3E). The up-regulating effects of cortisol omission could be repeated for the mouse *Vdr* gene, but the effects of short-term cortisol (2.4-fold) and IBMX (17.8- and 13.2-fold) treatment were completely opposite. This agrees with the observed difference in the time profile of the *Vdr* gene during differentiation. Finally, the expression of *GR* gene was 1.5- to 2-fold repressed after 4 and 8 h cortisol or IBMX stimulation and 1.4- to 2.5-fold weaker repressed after one and three days cortisol or IBMX omission (Fig. 3F). The mouse *Gr* gene showed similar effects. Interestingly, since cortisol is the activating ligand of GR, these data confirm the presence of the previously described auto-repression mechanism for the regulation of the *GR* gene also in adipose tissue [24]; [25].

In order to find out whether any difference in the combination of cortisol and IBMX may explain the differential time profile of the up-regulated genes, profiles for up-regulated nuclear receptor genes were obtained from the same experiments. All three genes showed different responses. A cortisol treatment of 4 and 8 h up-regulated *AR* expression 3.2- and 5.5-fold, while omitting the GR ligand led to 3.3-fold weaker increase after one day and 3.7-fold weaker increase after three days (Fig. 3G). Interestingly, IBMX reduced the gene 1.6-fold after 4 h, but had otherwise no effects. *PPARG* expression was up-regulated (2.3-fold) after 8 h cortisol treatment but repressed after IBMX stimulation for 4 h (1.7-fold) and 8 h (1.2-fold) (Fig. 3H). After one and three days the lack of cortisol lowered the activation of the gene 3-fold and 7.5-fold, respectively, while the omission of IBMX resulted only after three days in a 2-fold reduction in the up-regulation. The short-term down-regulation by IBMX and the effects of the omission of cortisol could be confirmed with the mouse *Pparg* gene. An initial repression by IBMX was also observed for the *LXRA* gene in human and mouse cells. After 8 h stimulation cortisol up-regulated the *LXRA* gene 2.1-fold, while lacking cortisol or IBMX for three days in the differentiation medium reduced the up-regulation of the gene 2.6- and 2.1-fold (Fig. 3I). In the mouse model, the IBMX repression persisted for the first 8 h, whereas in the human cells the effect reversed to a 1.9-fold up-regulation at this time point. The strong reduction in up-regulation of *Lxra* after three days without cortisol was observed in both species. For comparison, the strong GR target gene *TSC22D3* was up-regulated 22.3- and 26.5-fold after 4 and 8 h cortisol treatment and cortisol omission from the differentiation medium reduced its up-regulation 25.2- and 11.7-fold after one and three days (Fig. 3J). Similar but less potent effects were observed for the mouse *Tsc22d3* orthologue. Interestingly, both in the human and the mouse system also IBMX had an effect on *TSC22D3*/*Tsc22d3* gene expression: it was approximately 2-fold reduced after short-term treatment and also less repressed after omission of IBMX from the differentiation medium. This suggests a wider regulatory antagonism of the two compounds in the early up-regulation events during differentiation.

Taken together, a coherent cortisol- and IBMX-mediated repression of a few nuclear receptor genes is responsible for the first regulatory effects during early steps of differentiation. However, an initial divergence of the cortisol- and IBMX-mediated effects was observed among the up-regulated nuclear receptor genes: cortisol induced their expression levels, while IBMX lead to their repression. Such opposing effects would explain an initial flat signal, when both compounds are present in the full differentiation mix. This conflicting effect is observed to resolve at later time points in favor of the cortisol-mediated up-regulatory effects.

### The effect of the auto-repressive loop on GR-mediated effects

In the mouse system the role of GR in adipogenesis has already been extensively studied [26]; [27]. Moreover, the auto-repressive effect of glucocorticoids on the level of GR has been reported before in other cell types [24]; [25]; [28]. In the context of regulatory feed-back motifs such an interaction has been reported to result in an acceleration of the response and the maximal response level is reached early [29]. In this situation, the initial stimulation of the cells with cortisol is expected to lead to rapid GR-mediated effects on target genes, which are later tuned by the repressive effect of GR on its own transcription. Re-inspection of the time profile of the *GR* expression (Fig. 2) fits with a fast regulatory effect followed by dampening of the response, indicative of a fine-tuning of the regulatory effect. This is also reflected by the profiles of the majority of the repressed genes (Fig. 2) that reached their maximal repression level rapidly during the first 12 h and remain thereafter at a constant level or are less repressed.

**Figure 4:**
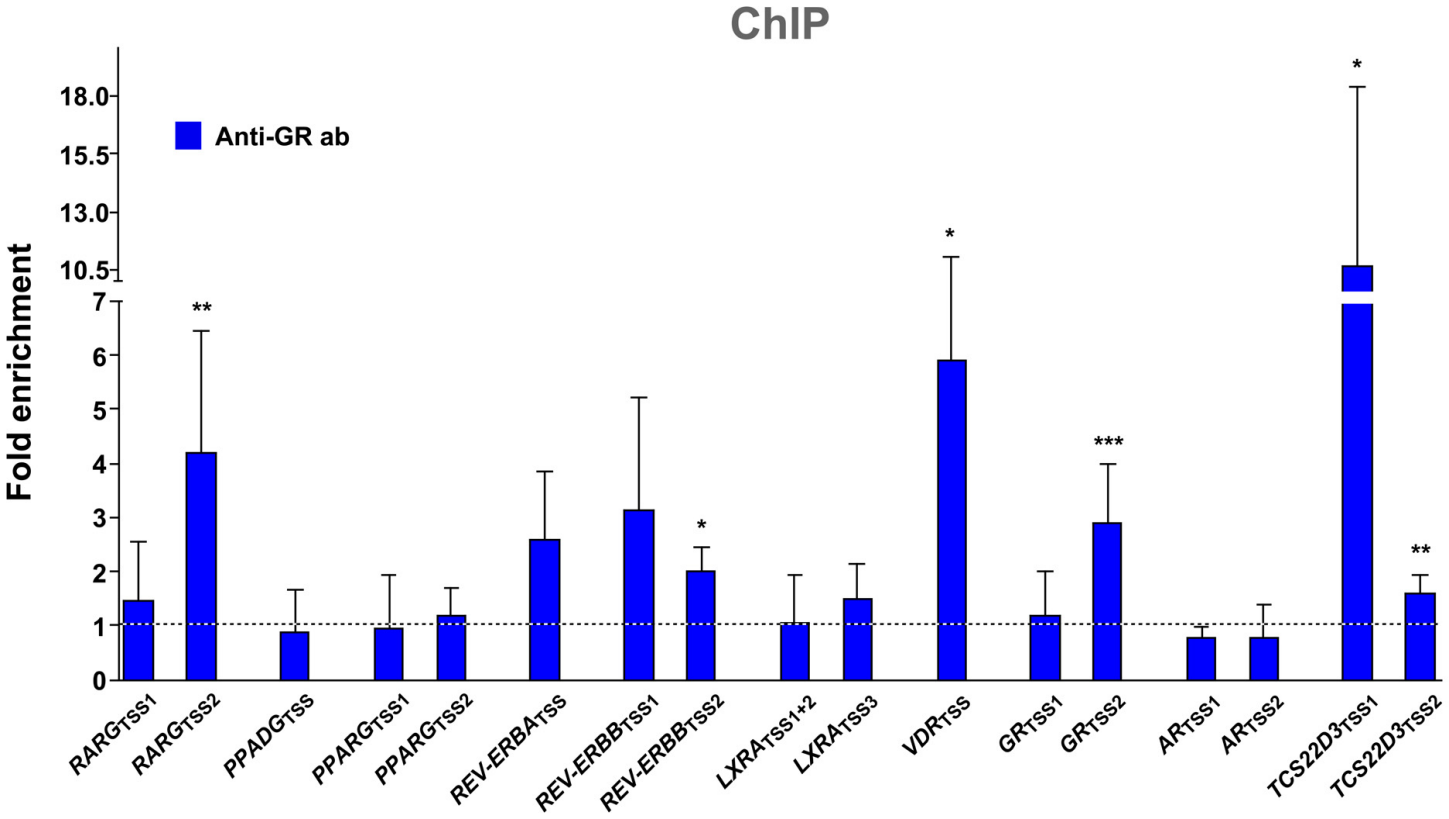
GR association with the TSS regions of nuclear receptor genes in undifferentiated human SGBS pre-adipocytes. ChIP assays were performed with chromatin extracts of untreated or 60 min 25 mM dexamathasone treated undifferentiated SGBS cells using an anti-GR antibody. Real-time quantitative PCR was performed using primers specific for the TSS regions of nine nuclear receptor genes and of the reference gene *TSC22D3*. Non-precipitated input chromatin served as reference and IgG-precipitated template as specificity control (data not shown). Fold induction of GR association was calculated. Columns represent the means of at least three biological repeats and the bars indicate standard deviations. Two-tailed paired Student's t-tests were performed to determine the significance of GR binding at 60 min in reference to undifferentiated pre-adipocytes (0 min) (* p<0.05, ** p<0.01, *** p<0.001).

In order to investigate, whether GR association was observed with the regulatory regions of early responding genes also in the human system, we performed ChIP assays with extracts from undifferentiated SGBS cells using anti-GR antibody (Fig. 4). Since primary GR target genes should show GR binding to their TSS regions (either directly or via looping from distal regulatory elements), we tested all early responding nuclear receptor genes and the *TSC22D3* reference gene. It should be noted that some of the genes have multiple TSS regions (for genomic view, see Fig. S6). GR binding to chromatin of SGBS cells that had been treated for 60 min with the synthetic GR ligand dexamethasone was compared with the association with chromatin of untreated cells. Non-specific antibody IgG was used as a negative control and provided similar background as that obtained from non-stimulated cells (data not shown). Both TSS regions of the reference gene *TSC22D3* showed statistically significant induction of GR binding as well as the chromatin regions *RARG*_TSS2_, *REV-ERBB*_TSS2_, *VDR*_TSS_ and *GR*_TSS2_, confirming four out of six early responding nuclear receptor genes, importantly also the auto-repression of *GR*. Increased association, although not statistically significant, was found with regions of the down-regulated genes *REV-ERBA*_TSS_ and *REV-ERBB*_TSS1_ and with the up-regulated gene *LXRA*_TSS3_, while the other regions (*RARG*_TSS1_, *PPARD*_TSS_, *PPARG*_TSS1_, *PPARG*_TSS2_, *LXRA*_TSS1+2_, *GR*_TSS1_, *AR*_TSS1_ and *AR*_TSS2_) showed no ligand-induced GR binding.

Since GR association with TSS regions is often the result of DNA looping of GR binding enhancer regions, we analyzed the only presently available ChIP-Seq dataset with anti-GR antibody from human lung cells [30] for the genomic regions 100 kB upstream and downstream of the TSS of the nine human nuclear receptor genes and the *TSC22D3* reference gene for potential GR binding (Fig. S6). Within the 200 kB genomic sequence around the *TSC22D3* gene TSS, 15 sites of GR association were identified, while the number of experimentally confirmed GR binding sites were 8 for the *REV-ERBA* gene, 4 for both the *RARG* and the *PPARG* gene, 3 for both the *VDR* and the *AR* gene, 2 for the *REV-ERBB* gene, 1 for both the *LXRA* and the *GR* gene and none for the *PPARD* gene. With the exception of the genes *PPARG* and *GR,* the number of GR binding sites correlates well with the increase of GR association with the respective gene's TSS.

In summary, our ChIP data provide further evidence for direct cortisol/GR-mediated regulation of eight nuclear receptor genes. For the set of repressed genes this association is observed at a very early 60 min time point in the SGBS cells, which is consistent with a fast regulatory mechanism associated with a negative auto-regulation.

## Discussion

In this study, human SGBS pre-adipocytes were used to investigate the regulation of nuclear receptor gene expressions during early steps of adipocyte differentiation. This is the first study in human adipocytes, where the regulation of an entire transcription factor family has been characterized with detailed time courses and with respect to different signaling pathways that initiate the adipogenesis. Furthermore, we provided a comparison to mouse 3T3-L1 cells that have been used to characterize this process previously [20]. Nuclear receptors are central regulators of metabolism and differentiation and due to the ligand-inducibility of many family members interesting therapeutic targets [6]; [7]. However, so far most studies on adipogenesis have been focused only on PPARγ, while far less attention was taken on other members of the nuclear receptor family.

In SGBS cells, quantitative PCR could detect the mRNA expression of 30 members of the nuclear receptor superfamily. When comparing pre-adipocytes with 12 days differentiated SGBS cells, 21 of these 30 nuclear receptor genes significantly changed their expression level indicating that the differentiation process resulted in major changes in the nuclear receptor transcriptional network. In a time course experiment a profound repression of six nuclear receptor genes characterized the initiation of differentiation and a time lag was observed for up-regulated nuclear receptor genes.

From the 15 down-regulated nuclear receptor genes in SGBS cells *RARG*, *PPARD*, *REV-ERBA*, *REV-ERBB*, *VDR* and *GR* show a response already in the early phase of adipogenesis. RARs are known to mediate the inhibition of adipocyte differentiation by their ligand all-*trans* retinoic acid [31] and similarly VDR has been shown to have a role in inhibiting adipogenesis in mouse 3T3-L1 cells [32]. However, the profile of the *VDR* gene shows a remarkable interspecies difference. In human adipocytes the *VDR* gene is constantly repressed with maximal 8-fold reduction at time point 12 h. In contrast, in 3T3-L1 cells the *Vdr* gene is first strongly up-regulated (up to 17-fold), but then at later time points approximately 2-fold down-regulated. This interspecies difference could lead to a differential response to VDR ligands and would be worth a more detailed investigation. Similarly, the strong down-regulation of the NR4A nuclear receptor subfamily members *NUR77*, *NURR1* and *NOR1* in SGBS cells, is in contrast to their reported up-regulation profile in the 3T3-L1 [20]–[22]. Another, less pronounced difference between SGBS and 3T3-L1 cells is the down-regulation of *PPARD* in human cells, while in mouse cells, consistent with the literature [33]; [34], *Ppard* is late up-regulated. Overall there is quite good correlation between the expression profiles of nuclear receptor genes in human SGBS cells and mouse 3T3-L1 cells. This applies also for the biphasic expression profile of the genes *GR*, *REV-ERBA* and *REV-ERBB* [20].

*REV-ERBA* induction by PPARγ activation with rosiglitazone has been shown in rat epididymal and perirenal adipose tissues *in vivo* as well as in 3T3-L1 cells *in vitro* [35]. Interestingly, another study reported that REV-ERBα inhibits the adipogenic program by repressing the expression of PPARγ [36]. Slightly reduced levels of *Rev-erba* were also observed, when rosiglitazone was omitted from the differentiation mix in 3T3-L1 cells but not in SGBS cells, again revealing an interspecies difference in the nuclear receptor regulation during adipogenesis. REV-ERBA is also known as one of the key genes in a circadian autoregulatory transcriptional feedback loop entraining central and peripheral oscillations [37]; [38]. A possible alternative interpretation of the early biphasic responses observed is that some components of the differentiation mix may induce circadian rhythmicity. In fact, cortisol and T_3_ levels are regulated in a circadian fashion [39] and cAMP signaling has been shown to regulate the expression of clock genes [40]. In mice 24 h light/dark cycles were shown to induce a circadian rhythm in the expression of many members of the nuclear receptor superfamily in metabolic tissues, such as white adipocyte tissue [41]. The circadian expression pattern of the *REV-ERBs* and other clock genes in adipocytes was reported previously [42]. The connection between a circadian regulatory mechanism and adipogenic differentiation program merits further investigations in future.

The observation of a down-regulation of many members of the nuclear receptor superfamily during adipogenesis fits with other studies that have shown that a number of transcription factors attenuate adipogenesis and serve to function as molecular switches in controlling the fate of the progenitor cells (for a review see [2]).

The SGBS and the 3T3-L1 cell model as well as primary human adipocytes consistently indicate strong up-regulation of the genes *PPARG* and *LXRA* during adipogenesis. *PPARG* up-regulation is well known, but the even stronger increase of *LXRA* expression has not received much attention so far in the human system. Interestingly, both PPARγ and LXRα are known as central transcription factors controlling genes needed for lipid metabolism and transport [43], which is important for the function of differentiated adipocytes. This is in accordance with our observation that *LXRA* expression is up-regulated later in adipogenesis. Moreover, our comparison of undifferentiated and differentiated SGBS cells indicated up-regulation of the genes *THRA*, *THRB*, *RXRA* and *AR*. Only the up-regulation of *AR* is observed in early phases of adipogenesis. Its increase as well as that of *RXRA* is also detectable in long-term differentiated (30 days) primary human adipocytes [23]. Of note, we could not detect *Ar* expression in 3T3-L1 cells and also other studies have reported low expression of the receptor in mouse cells [20], while studies in humans have reported an anti-adipogenic role for this nuclear receptor [44]. Interestingly T_3_, the ligand of the gene products of *THRA* and *THRB*, TRα and TRβ, is a component of the differentiation mix. This fits with the recent observation that adipogenesis was impaired in cells expressing loss-of-function mutants of *THRA* and *THRB* [45].

The expression profiles indicate that the cellular responsiveness shifts from the micronutrient responsive receptors VDR and RAR to the hormone responsive receptors AR and TR, all of which have been reported to control adipogenesis and lipid accumulation [31]; [32]; [44]; [46]. This is accompanied by the strong up-regulation of PPARγ and LXRα. Overall, our observation on the time-resolved expression changes of nuclear receptor genes in human SGBS cells is in good accordance with the study of Fu *et al.* [20], where similar time-resolved expression profiles were measured for the 49 mouse members of the nuclear receptor superfamily in 3T3-L1 cells. However, for follow-up investigations of the responsible signaling pathways, we found human SGBS cells better suited than 3T3-L1 cells, because in the human cells adipogenesis is performed in the absence of FBS. Therefore, we were able to test each of the five mix components individually without the risk of a possible interference with undefined serum components.

The characterization of signaling pathways that lead to the initiation of the early regulatory cascades revealed that cortisol and IBMX are responsible for the vast majority of the observed changes in human pre-adipocytes. This fits with a recent study in mouse cells, where cortisol was also shown to be the predominant inducer of differentiation [47]. With the other differentiation mix components, T_3_, insulin and rosiglitazone, only minor effects were observed at early time points. Cells that were stimulated for 48 h with cortisol followed by IBMX treatment differentiate normally, whereas cells that receive the two compounds in the inverse order failed to differentiate. Interestingly, our results show that the early repression effects and up-regulation differ in the integration of signaling pathways. The six repressed genes were responsive to both cortisol and IBMX in a coherent manner. In contrast, antagonism of these pathways was observed for all three up-regulated genes, fitting with an initial flat signal, when both compounds are present in the full differentiation mix.

Glucocorticoids are endogenous stress-induced hormones that are produced under the control of the hypothalamic-pituitary-adrenal axis. They are highly lipophilic and like other nuclear receptor ligands diffuse through the plasma membrane and bind to cytosolic GR being complexed with heat-shock proteins and co-chaperones [48]. In several cell lines and tissues the administration of glucocorticoids results in GR down-regulation [24]; [25]. This down-regulation has been attributed to reduced transcription of the *GR* gene as well as decreased mRNA and protein stability [49]–[51]. In bacteria and yeast negative auto-regulation has been described to lead to rapid response times and noise filtering [52]; [53]. In accordance with that, the GR/cortisol-dependent expression profiles of nuclear receptor genes show an early maximal response within the first 24 h, followed by a lower level of repression at later time points. We showed that ligand-activated GR associates with the TSS regions of *RARG*, *REV-ERBB*, *VDR* and the TSS of the *GR* gene itself. Moreover, based on the lung cell ChIP-Seq data GR associates to three regions of the *RARG* gene, to eight regions of the *REV-ERBA* gene, to two in the *REV-ERBB* gene, to three in the *VDR* gene and to one in the *GR* gene. Both data sets indicate that the four nuclear receptor genes are primary GR target genes [30].

In conclusion, adipocyte differentiation is a process, in which many members of the nuclear receptor superfamily change their mRNA expression. The actions of cortisol and IBMX were shown to interfere and to mediate the majority of the initial gene regulatory effects. Interestingly, both compounds converged to repress several nuclear receptors including the anti-adipogenic genes *RARG*, *REV-ERBA* and *VDR*. In contrast, up-regulation of nuclear receptor genes showed a time lag, which was attributed for the three studied genes to initial antagonism of the effects of IBMX and cortisol. Our results place GR and its ligand cortisol as central regulatory factors controlling early regulatory events in human adipogenesis.

## Methods

### Cell culture and differentiation

SGBS cells [16] were cultured in Dulbecco's modified Eagle's medium (DMEM)/Nutrient Mix F12 (Gibco, Paisley, UK) containing 8 mg/l biotin, 4 mg/l pantothenate, 0.1 mg/ml streptomycin and 100 U/ml penicillin (OF medium) supplemented with 10% FBS in a humidified 95% air/5% CO_2_ incubator. 3T3-L1 cells were cultured in DMEM supplemented with 10% FBS, 2 mM L-glutamine, 0.1 mg/ml streptomycin and 100 U/ml penicillin. The SGBS cells were seeded into culture medium flasks or plates, which were coated with a solution of 10 µl/ml fibronectin and 0.05% gelatine in phosphate-buffered saline (PBS). Confluent cells were treated with the respective compounds. For stimulation experiments 25 nM dexamethasone (diluted in DMSO), 685 nM cortisol (also called hydrocortisone, diluted in EtOH) or 500 µM IBMX (diluted in DMSO) were used.

Differentiating SGBS cells were kept in 3FC medium (OF media supplemented with 0.01 mg/ml human transferrin (Sigma-Aldrich)), 100 nM T_3_, 685 nM cortisol and 20 nM insulin (Sigma-Aldrich). The differentiation rate was increased by the addition of 500 µM IBMX and 100 nM rosiglitazone (Cayman Chemical, Ann Arbor, USA), referred to as Quick-diff medium. Cells were incubated for the first four days in Quick-diff medium. Thereafter 3FC medium was used and changed every 96 h. SGBS cells differentiate within 10-12 days as determined by microscopic analysis. With 3T3-L1 cells (ATCC, CL-173) the identical differentiation procedure as for SGBS cells was performed, but the medium contained FBS and was changed every 48 h. 3T3-L1 cells differentiate within 6 days. For both cellular models the grade of differentiation was determined by Oil red O staining for fat vacuoles (Fig. S7).

### RNA extraction and real-time quantitative PCR

Total RNA was extracted using Tri Reagent (Sigma-Aldrich) according to the manufacturer's protocol and cDNA synthesis was performed for 30 min at 55°C using 1 µg of total RNA as a template and 100 pmol oligodT_18_ primers. Real-time quantitative PCR was performed using a LightCycler® 480 System (Roche Diagnostics). For human cDNA templates 4 pmol human-specific primers primers (Table S1), 4 µl cDNA template, 1 U FastStart Taq polymerase (Fermentas, Vilnius, Lithuania), 2.75 mM MgCl_2_ and SybrGreen (Invitrogen) were used in a total volume of 10 µl. In the PCR reaction the DNA templates were pre-denaturated for 10 min at 95°C, followed by amplification steps cycles of 30 s denaturation at 95°C, 30 s annealing at primer-specific temperatures (Table S1), 30 s elongation at 72°C and a final elongation for 5 min at 72°C. For mouse cDNA templates mouse-specific primers (Table S2) and the Maxima™ SYBR Green/ROX qPCR Master Mix (Fermentas) was used. The amplification steps in the PCR reaction were each only 15 s. Fold inductions were calculated using the formula 2^-(ΔΔCt)^, where ΔΔCt is ΔCt_(treatment)_ - ΔCt_(solvent)_, ΔCt is Ct_(target gene)_ - Ct_(*RPL13A*)_ and the Ct is the cycle, at which the threshold is crossed. Basal expression levels were calculated using the formula 2^-(ΔCt)^. PCR product quality was monitored using post-PCR melt curve analysis.

### ChIP assay

Nuclear proteins were cross-linked to DNA by adding formaldehyde directly to the medium to a final concentration of 1% for 8 min at room temperature. Cross-linking was stopped by adding glycine to a final concentration of 0.125 M and incubating for 5 min at room temperature on a rocking platform. The medium was removed and the cells were washed twice with ice-cold PBS. The cells were then collected in lysis buffer (1% SDS, 10 mM EDTA, protease inhibitors, 50 mM Tris-HCl, pH 8.1) and the lysates were sonicated by a Bioruptor UCD-200 (Diagenode, Liege, Belgium) to result in DNA fragments of 200 to 1000 bp in length. Cellular debris was removed by centrifugation and the lysates were diluted 1∶10 in ChIP dilution buffer (0.01% SDS, 1.1% Triton X-100, 1.2 mM EDTA, 167 mM NaCl, protease inhibitors, 16.7 mM Tris-HCl, pH 8.1). Chromatin solutions were incubated overnight at 4°C with rotation with 7.5 µl of anti-GR antibody (Santa Cruz Biotechnologies, sc-8992) or 1 µl control IgG (both from Upstate Biotechnology, Lake Placid, NY, USA). The immuno-complexes were collected with 60 µl of protein A agarose slurry (Upstate Biotechnology) for 1 h at 4°C with rotation. Non-specific background was removed by incubating the Protein A agarose bead slurry overnight at 4°C with rotation in the presence of BSA (10 mg/ml). The beads were precipitated by centrifugation for 1 min at room temperature with 100x g and washed sequentially for 3 min by rotation with 1 ml of the following buffers: low salt wash buffer (0.1% SDS, 1% Triton X-100, 2 mM EDTA, 150 mM NaCl, 20 mM Tris-HCl, pH 8.1), high salt wash buffer (0.1% SDS, 1% Triton X-100, 2 mM EDTA, 500 mM NaCl, 20 mM Tris-HCl, pH 8.1) and LiCl wash buffer (0.25 M LiCl, 1% Nonidet P-40, 1% sodium deoxycholate, 1 mM EDTA, 10 mM Tris-HCl, pH 8.1). Finally, the beads were washed twice with 1 ml TE buffer (1 mM EDTA, 10 mM Tris-HCl, pH 8.1). The immuno-complexes were then eluted by adding 500 µl of elution buffer (25 mM Tris-HCl, pH 7.5, 10 mM EDTA, 0.5% SDS) and incubating for 30 min at 65°C. The cross-linking was reversed and the remaining proteins were digested by adding 2.5 µl of proteinase K (Fermentas) to a final concentration of 80 µg/ml and incubating overnight at 64°C. The DNA was recovered by phenol/chloroform/isoamyl alcohol (25∶24∶1) extractions and precipitated with 0.1 volume of 3 M sodium acetate, pH 5.2, and 2 volumes of ethanol using glycogen as carrier. Immuno-precipitated chromatin DNA was then used as a template for real-time quantitative PCR.

### PCR of chromatin templates

Real-time quantitative PCR of ChIP templates was performed using chromatin-region specific primers (Table S3) and Maxima™ SYBR Green/ROX qPCR Master Mix in a total volume of 10 µl in a LightCycler® 480 System. For the TSS regions of *RARG*, *PPARD*, *PPARG* and *LXRA* the use of 1 U FastStart Taq polymerase, 1.7 mM MgCl_2_ and SybrGreen improved PCR efficiency. The PCR cycling conditions were: pre-incubation for 10 min at 95°C, 50 cycles of 20 s denaturation at 95°C, 20 s annealing at primer-specific temperatures (Table S3), 20 s elongation at 72°C and a final elongation for 5 min at 72°C. The PCR products were also resolved on 2% agarose gels to control correct product size. Relative association of chromatin-bound protein or histone modifications were calculated using the formula 2^-(ΔCt)^*100, where ΔCt is Ct_(output)_ - Ct_(input)_, output is the immuno-precipitated DNA and input is the purified genomic DNA from starting material of the ChIP assay. In PCR 1% of input sample was used and later on corrected by the respective dilution factors.

## Supporting Information

Table S1 Real-time quantitative PCR primers for human genes. Primer sequence, product size and annealing temperature used for gene-specific real-time quantitative PCR are listed.
(0.13 MB DOC)

Table S2 Real-time quantitative PCR primers for mouse genes. Primer sequence, product size and annealing temperature used for gene-specific real-time quantitative PCR are indicated.
(0.08 MB DOC)

Table S3 Genomic PCR primers. Sequence and location (relative to TSS) of the primer pairs used to detect genomic regions of the nine nuclear receptor genes and the control region of the *TSC22D3* gene. The genomic location is based on assembly NCBI36/hg18.
(0.10 MB DOC)

Figure S1 Nuclear receptor expression profiling during human SGBS cell differentiation. Real-time quantitative PCR was performed in order to determine mRNA expression of nuclear receptor genes in relation to the housekeeping gene *RPL13A* at indicated time points of SGBS differentiation. The 21 of the 30 in SGBS cells expressed nuclear receptor genes that are not shown in Fig. 2 are displayed. Three genes were late activated (A), five were late repressed (B) and 13 showed a mixed or a modest response (C). Columns represent the means of at least three biological repeats and the bars indicate standard deviations. Two-tailed paired Student's t-tests were performed to determine the significance of the mRNA level changes in reference to undifferentiated pre-adipocytes (* p<0.05, ** p<0.01, *** p<0.001).
(0.08 MB PDF)

Figure S2 Nuclear receptor mRNA expression in undifferentiated and differentiated mouse 3T3-L1 cells. Real-time quantitative PCR with gene-specific primers was used to determine the mRNA expression levels of eight selected nuclear receptor genes in relation to the housekeeping gene *Rplp0* in undifferentiated (blue triangles) or 6 days differentiated (red circles) 3T3-L1 cells (*Ar* is not expressed). Fold changes were calculated in reference to undifferentiated cells. Data points represent the means of three biological repeats and the bars indicate standard deviations.
(0.12 MB PDF)

Figure S3 Nuclear receptor expression profiling during mouse 3T3-L1 cell differentiation. Real-time quantitative PCR was performed in order to determine mRNA expression of eight selected nuclear receptor genes in relation to the housekeeping gene *Rplp0* at indicated time points of 3T3-L1 differentiation. Four genes were mainly activated (A) and four genes were mainly repressed (B). Columns represent the means of at least three biological repeats and the bars indicate standard deviations. Two-tailed paired Student's t-tests were performed to determine the significance of the mRNA level changes in reference to undifferentiated pre-adipocytes (* p<0.05, ** p<0.01, *** p<0.001).
(0.05 MB PDF)

Figure S4 Nuclear receptor mRNA expression in primary human pre-adipocytes and adipocytes. Raw data for primary human pre-adipocytes (blue triangles) or 30 days differentiated (red circles) adipocytes were obtained from the GSE1657 dataset of the public microarray repository NCBI Gene Expression Omnibus (www.ncbi.nlm.nih.gov/geo). The probe values were background corrected using the gc-rma full model, normalized using quantile normalization and summarized to gene expression values using median polish. The mRNA expression levels of all 48 nuclear receptor genes were compared and fold changes were calculated. Data points represent the means of three biological repeats and the bars indicate standard deviations. For the gene *ESRRB* the Affymetrix HG-U133A array did not contain any specific probe (see University of Michigan BrainArray project, http://brainarray.mbni.med.umich.edu/brainarray/default.asp). The horizontal line indicates the estimated threshold of specific expression.
(0.63 MB PDF)

Figure S5 Effects of individual differentiation medium compounds on nuclear receptor gene expression during early human SGBS and mouse 3T3-L1 cell differentiation. Real-time quantitative PCR was performed in order to determine mRNA expression of nine selected nuclear receptor genes in relation to the housekeeping genes *RPL13A* and *Rplp0* in SGBS and 3T3-L1 cells, respectively. The cells were either stimulated for 4 h with either T3 (T), cortisol (C), insulin (I), rosiglitazone (R) or IBMX (IB) or they were differentiated for 24 h with full differentiation medium lacking the indicated compounds. Columns represent the means of at least three biological repeats and the bars indicate standard deviations. Two-tailed paired Student's t-tests were performed to determine the significance of the mRNA level changes in reference to undifferentiated pre-adipocytes (for 4 h treatments) or in reference to full differentiation medium (for 24 h treatments) (* p<0.05, ** p<0.01, *** p<0.001).
(0.08 MB PDF)

Figure S6 Genomic structure of nuclear receptor genes. The UCSC genome browser was used to display the genomic regions +/− 100 kB to the TSS of nine nuclear receptor genes and the reference gene *TSC22D3*. GR association data to these regions were obtained from Reddy et al.
(3.14 MB PDF)

Figure S7 Human and mouse pre-adipocytes and adipocytes. SGBS human pre-adipocytes (top left) were differentiated to adipocytes (top right) within 11 days and 3T3-L1 mouse pre-adipocytes (bottom left) were differentiated within 6 days (bottom right). Accumulation of lipid droplets was visualized by Oil Red O staining.
(0.17 MB PDF)
