# Supplementary material for: Time-Resolved Expression Profiling of the Nuclear Receptor Superfamily in Human Adipogenesis
Source: PLoS One. 2010 Sep 27;5(9):e12991. doi: 10.1371/journal.pone.0012991 (PMC2946337; doi:10.1371/journal.pone.0012991)
Supplement: Table S1 — Real-time quantitative PCR primers for human genes. Primer sequence, product size and annealing temperature used for gene-specific real-time quantitative PCR are listed. (0.13 MB DOC) [file pone.0012991.s001.doc]

**Table S1: Real-time quantitative PCR primers for human genes.** Primer sequence, product size and annealing temperature used for gene-specific real-time quantitative PCR are listed.

| **Gene** | **Primer pairs (5'-3')** | **Product size (bp)** | **Annealing temperature (°C)** |
| --- | --- | --- | --- |
| *RPL13A* | CCTGGAGGAGAAGAGGAAAGAGA  TTGAGGACCTCTGTGTATTTGTCAA | 125 | 58-62 |
| *THRA*  *(NR1A1)* | CGACGCCATCTTTGAACTG  GTACGCCTCCTGACTCTTC | 147 | 58 |
| *THRB*  *(NR1A2)* | GTCCTGCTGATGTCTTCAG  GTCAGTCTAATCCTCGAACAC | 266 | 60 |
| *RARA*  *(NR1B1)* | ACAGCTCAGAACAACGTGTC  CTTCGCAGGTCAGTAATCTTC | 522 | 60 |
| *RARB*  *(NR1B2)* | CATCTGCTTAATCTGTGGAGAC  CACGAGTGGTGACTGACTG | 369 | 60 |
| *RARG*  *(NR1B3)* | CTCACAGACCTTGTCTTTGC  CTGGAATCTCCATCTTCAGAG | 297 | 60 |
| *PPARA*  *(NR1C1)* | TGCTGTCTTCTGTGATGAAC  TCTGAGCACATGTACAATAC | 268 | 58 |
| *PPARD*  *(NR1C2)* | CTCTATCGTCAACAAGGACG  GTCTTCTTGATCCGCTGCAT | 383 | 58 |
| *PPARG*  *(NR1C3)* | CGACCAAGTAACTCTCCTCA  GTTCCGTGACAATCTGTCTG | 409 | 58 |
| *REV-ERBA*  *(NR1D1)* | AGGACCAGACAGTGATGTTC  CTTCTCGGAATGCATGTTGTTC | 322 | 60 |
| *REV-ERBB*  *(NR1D2)* | CAGTGAAAGAAGTGGTGGAATTTG  GATCTGGCAACTTTAGAAGCAG | 446 | 58 |
| *RORA*  *(NR1F1)* | GATGTATTTTGTGATCGCAGC  CATTTCTGTAATCGACAGTGTTG | 230 | 58 |
| *RORB*  *(NR1F2)* | GTTCTGATGACCTAGTGAATG  CTTAAATACCTGCAGCTTCTC | 292 | 58 |
| *RORG*  *(NR1F3)* | GTAGAACAGCTGCAGTACAATC  CTTCCAGGTCACTTGGAC | 250 | 58 |
| *LXRA*  *(NR1H3)* | CGGGCTTCCACTACAATGTT  TCAGGCGGATCTGTTCTTCT | 212 | 58 |
| *LXRB*  *(NR1H2)* | CTGCAGGTGGAGTTCATCAAC  ACGTCCCAGATCTCCGACAG | 343 | 58 |
| *FXR*  *(NR1H4)* | GAAATAGTGGTATCTCTGATG  CAGCGTGGTGATGATTGAAT | 287 | 58 |
| *VDR*  *(NR1I1)* | AGATGACCCTTCTGTGACCC  AGCTTCTTCAGTCCCACCTG | 332 | 58 |
| *PXR*  *(NR1I2)* | CAAATCTGCCGTGTATGTGG  ATCTGAGCGTCCATCAGCTC | 361 | 60 |
| *CAR*  *(NR1I3)* | ATGAGGAAAGACATGATACTG  CTTGAGAAGGGAGATCTGG | 359 | 60 |
| *HNF4A*  *(NR2A1)* | CTTGGAGGACTACATCAACGAC  CATCAGGTGAGGGTGCAG | 228 | 58 |
| *HNF4G*  *(NR2A2)* | CAAGTGCAGATCGGTTTGGAG  CAGGAGTTGAGATCTGGTCTG | 321 | 60 |
| *RXRA*  *(NR2B1)* | GCTGGAATGAGCTGCTCATC  GGTACTTGTGCTTGCAGTAG | 314 | 60 |
| *RXRB*  *(NR2B2)* | CTGGATGATCAGGTCATATTG  CAGATGCTCTAGACACTTAAG | 428 | 60 |
| *RXRG*  *(NR2B3)* | CTTTGACAGAGTCCTAACTGAG  GTCTCCAACATCTCCATGAG | 388 | 60 |
| *TR2*  *(NR2C1)* | CTTCAGTCCAGATCATCCAAG  GTCACAGTTTTCAAATGCTGTGAC | 301 | 60 |
| *TR4*  *(NR2C2)* | CTGCAGGAGTTCTGTAACAG  GAGTAGGTTTTCTGAACATAGTC | 178 | 58 |
| *TLL*  *(NR2E1)* | CTTGGAGAGAACTGTTTGTTCTAG  CTGGTATGGATGTAGCTGTTG | 333 | 60 |
| *PNR*  *(NR2E3)* | CGTGGAGTGAACTCTTTCTC  GTTTTCCAGAGTGCTTGGAAAC | 506 | 58 |
| *COUP-TFI*  *(NR2F1)* | CATCGTGCTGTTCACGTCAG  CACTGGATGGACATGTAAGG | 293 | 60 |
| *COUP-TFII*  *(NR2F2)* | CCACATATAACACTTAACTTCC  CTAAGATACTGAATCAATCACG | 354 | 60 |
| *EAR2*  *(NR2F6)* | GTGGCTTTCATGGACCAG  CAGCATGTCTCTGATCAGTG | 344 | 60 |
| *ESRA*  *(NR3A1)* | CTGGAGTGTACACATTTCTGTC  CTTCATGCTGTACAGATGCTC | 220 | 58 |
| *ESRB*  *(NR3A2)* | CTCCAGATCTTGTTCTGGACAG  CATGTTGAGCAGATGTTCCATG | 367 | 58 |
| *ESRRA*  *(NR3B1)* | GTCTGACCAGATGTCAGTAC  CATCTTCGATGTGCACAGAG | 271 | 58 |
| *ESRRB*  *(NR3B2)* | CATGCACAAACTCTTCCTGGAGATG  CATCATGGCTTGACATTCTTTCATC | 281 | 60 |
| *ESRRG*  *(NR3B3)* | GACGAAGACCAGTCCAAATTAG  GCAGTGTCATCAGCATCTTG | 267 | 60 |
| *GR*  *(NR3C1)* | GGAACTTACACCTGGATGAC  GTAACTCAGAGGAAACATACAG | 206 | 58 |
| *MR*  *(NR3C2)* | GAGATCGTACAAACATACGAACAG  GTAGAAGCAGAATTCCAGCAG | 384 | 60 |
| *PR*  *(NR3C3)* | GTTTGAGGAGATGAGGTCAAG  GTGCAGCAATAACTTCAGACATC | 226 | 60 |
| *AR*  *(NR3C4)* | CTTCACCAATGTCAACTCCAG  CAAAAGTGAACTGATGCAGCTC | 376 | 60 |
| *NUR77*  *(NR4A1)* | CAGCTTGCTTGTCGATGTC  GTGTCCATGAAGATCTTGTCAATG | 302 | 58 |
| *NURR1*  *(NR4A2)* | CTGACTATCAAATGAGTGGAGATG  GTCGATGTTCATATTCTGCAAGTTG | 325 | 58 |
| *NOR1*  *(NR4A3)* | GTCAGCACTGAGCATGATC  GAAGGTGGAGACACCAAG | 230 | 58 |
| *SFI*  *(NR5A1)* | CTCAAGTTCATCATCCTCTTCAG  TTGCAGCATTTCGATGAGCAG | 260 | 58 |
| *LRH1*  *(NR5A2)* | CATGAGTCATGCACAGGAG  GTTCAGGTGCTTGTAGTAGAG | 288 | 58 |
| *GCNF*  *(NR6A1)* | CTACCTCTATCACAAGTTCCATC  GAATGCAGCACCACCTTAAAG | 296 | 58 |
| *DAX1*  *(**NR0B1)* | CTGGAGTCTGAACATCAGTAC  CAGTTCAGCAATGACATTGGCA | 237 | 60 |
| *SHP*  *(NR0B2)* | CATACTCAAGAAGATTCTGCTG  GATGTCAACATCTCCAATGATAG | 390 | 60 |
| *TSC22D3* | GGATCTGGTGAAGAATCATC  ACAGGGTGTTCTCACGCTC | 115 | 60 |
