# Supplementary material for: Time-Resolved Expression Profiling of the Nuclear Receptor Superfamily in Human Adipogenesis
Source: PLoS One. 2010 Sep 27;5(9):e12991. doi: 10.1371/journal.pone.0012991 (PMC2946337; doi:10.1371/journal.pone.0012991)
Supplement: Table S2 — Real-time quantitative PCR primers for mouse genes. Primer sequence, product size and annealing temperature used for gene-specific real-time quantitative PCR are indicated. (0.08 MB DOC) [file pone.0012991.s002.doc]

**Table S2: Real-time quantitative PCR primers for mouse genes.** Primer sequence, product size and annealing temperature used for gene-specific real-time quantitative PCR are indicated.

| **Gene** | **Primer pairs (5'-3')** | **Product size (bp)** | **Annealing**  **temperature (°C)** |
| --- | --- | --- | --- |
| *Rplp0* | AGATTCGGGATATGCTGTTGGC  TCGGGTCCTAGACCAGTGTTC | 109 | 58-62 |
| *Rarg*  *(Nr1b3)* | CCATGCTTTGTATGCAATGACA  TTCTGAATGCTGCGTCTGAAG | 94 | 58 |
| *Pparg*  *(Nr1c3)* | CAAGAATACCAAAGTGCGATCAA  GAGCTGGGTCTTTTCAGAATAATAAG | 68 | 58 |
| *Ppard*  *(Nr1c2)* | GCCTCGGGCTTCCACTAC  AGATCCGATCGCACTTCTCA | 89 | 58 |
| *Rev-erba*  *(Nr1d1)* | TTCGGGAGGTGGTAGAGTTTGC  TGTCTGGTCCTTCACGTTGAACA | 142 | 58 |
| *Rev-erbb*  *(Nr1d2)* | GTTCACAGCAGTTGTTCTGGTA  GCCAAGAGTTCCTCAGAGTG | 207 | 58 |
| *Lxra*  *(Nr1h3)* | CTCAATGCCTGATGTTTCTCCT  TCCAACCCTATCCCTAAAGCAA | 150 | 58 |
| *Vdr*  *(Nr1i1)* | CATAAAGTTCCAGGTGGGGC  GGCAGCGGATGTAGGTCTG | 169 | 58 |
| *Gr*  *(Nr3c1)* | ATGTATGACCAATGTAAACACA  GCTCTTCAGACCTTCCTTAG | 131 | 58 |
| *Ar*  *(Nr3c4)* | GTTGGCGGTCCTTCACTAATGTC  CATGCGGTACTCATTGAAAACCA | 76 | 58 |
| *Tsc22d3* | GGGCAGAGATGGGAGAGATTGA  CTGTGCACAAAGCCATGCATT | 75 | 58 |
