# Supplementary material for: Time-Resolved Expression Profiling of the Nuclear Receptor Superfamily in Human Adipogenesis
Source: PLoS One. 2010 Sep 27;5(9):e12991. doi: 10.1371/journal.pone.0012991 (PMC2946337; doi:10.1371/journal.pone.0012991)
Supplement: Table S3 — Genomic PCR primers. Sequence and location (relative to TSS) of the primer pairs used to detect genomic regions of the nine nuclear receptor genes and the control region of the TSC22D3 gene. The genomic location is based on assembly NCBI36/hg18. (0.10 MB DOC) [file pone.0012991.s003.doc]

**Table S3: Genomic PCR primers.** Sequence and location (relative to TSS) of the primer pairs used to detect genomic regions of the nine nuclear receptor genes and the control region of the *TSC22D3* gene. The genomic location is based on assembly NCBI36/hg18.

| **Gene**  **TSS variant** | **Location** | **Chr.**  **strand** | **Genomic location** | | | **Primer sequence (5’-3’)** |
| --- | --- | --- | --- | --- | --- | --- |
|  |  |  | **Chr.** | **start** | **end** |  |
| *RARG*_TSS1_ | -236 to -156 | - | 12 | 51912522  51912462 | 51912542  51912482 | GACACTGACCTCCTCTGCCAG  TCTCTGCCCTTCCCATTTGCT |
| *RARG*_TSS2_ | -284 to -83 | - | 12 | 51900683  51900482 | 51900701  51900500 | AGCATTCTCCCGAGCAAGG  GCACCGAGATGAGCAAAGC |
| *PPARD*_TSS_ | -100 to +91 | + | 6 | 35418212  35418384 | 35418231  35418403 | CAGGAGGCGTGGTGATTGGC  TCCCGCAGGCTCCGCAGAAT |
| *PPARG*_TSS1_ | -245 to -168 | + | 3 | 12304102  12304160 | 12304120  12304179 | GATCCCTCCTCGGAAATGG  GCTACCTGGTGTCGTTTGCT |
| *PPARG*_TSS2_ | +81 to +139 | + | 3 | 12368081  12368120 | 12368102  12368139 | TCCATGCTGTTATGGGTGAAAC  GGAATCGCTTTCTGGGTCAA |
| *REV-ERBA*_TSS_ | -81 to +72 | - | 17 | 35510561  35510428 | 35510581  35510446 | TACAAATCCCGACAGTCTTGT  CCAGCAGCCCAGGGTTCC |
| *REV-ERBB*_TSS1_ | -196 to -98 | + | 3 | 23961558  23961638 | 23961576  23961656 | AAGAGGTTTACGGCACGGA  GCTACCCTTTTCTCCTGCG |
| *REV-ERBB*_TSS2_ | +178 to +339 | + | 3 | 23962793  23962936 | 23962811  23962954 | CCTGGGGAGGCTGGTAGC  GGGAGTCGCTCGGGATCG |
| *LXRA*_TSS1-2_ | +114 to +194 | + | 11 | 47236186  47236246 | 47236205  47236265 | ACCATCCTCTTCTCCCAGCA  CAGACCCACCAAATCCCCAG |
| *LXRA*_TSS3_ | -40 to +59 | + | 11 | 47226984  47227064 | 47227003  47227083 | ACGCACCCGTAAGGACACAC  ACGCAATCCCACCGAGACTG |
| *VDR*_TSS_ | -14 to +111 | - | 12 | 46585078  46584971 | 46585096  46584989 | GGTGCTGGGCTGTCTCTGC  GAGACCCCCTTTCCCGCTG |
| *GR*_TSS1_ | -17 to +129 | - | 5 | 142795269  142795143 | 142795288  142795163 | TCCTCCTATTGTGTGGTCAG  CAGCAGAAGTGAAAGTGATTC |
| *GR*_TSS2_ | +95 to +182 | - | 5 | 142763335  142763266 | 142763353  142763283 | CCTCCTCCATTTTGCGAGC  GTCTCCTTCCACCCACAG |
| *AR*_TSS1_ | -29 to +132 | + | X | 66680569  66680710 | 66680588  66680729 | CATTTGCTCTCCACCTCCCA  GTGCGTCCCTTCGGCTCCTG |
| *AR*_TSS2_ | -72 to +91 | + | X | 66705335  66705479 | 66705356  66705498 | TTCCCAACCCACTGTGTATTGC  CAAAGCCAGCCTCCAGCAGC |
| *TSC22D3*_TSS2_ | -1 to +78 | - | X | 106846930  106846871 | 106846949  106846890 | CGAGTCTGGGTTGGACTGG  TGGAGTTGAAGGGAAGTGAC |
| *TSC22D3*_TSS3_ | -258 to -195 | - | X | 106846605  106846562 | 106846626  106846582 | CTTCTCTTCTCTGCTTGGAG  GCCAGCGGTTACCTGTTGT |
