# Supplementary figures and images for: Time-Resolved Expression Profiling of the Nuclear Receptor Superfamily in Human Adipogenesis
Source: PLoS One. 2010 Sep 27;5(9):e12991. doi: 10.1371/journal.pone.0012991 (PMC2946337; doi:10.1371/journal.pone.0012991)

# Quantitative PCR

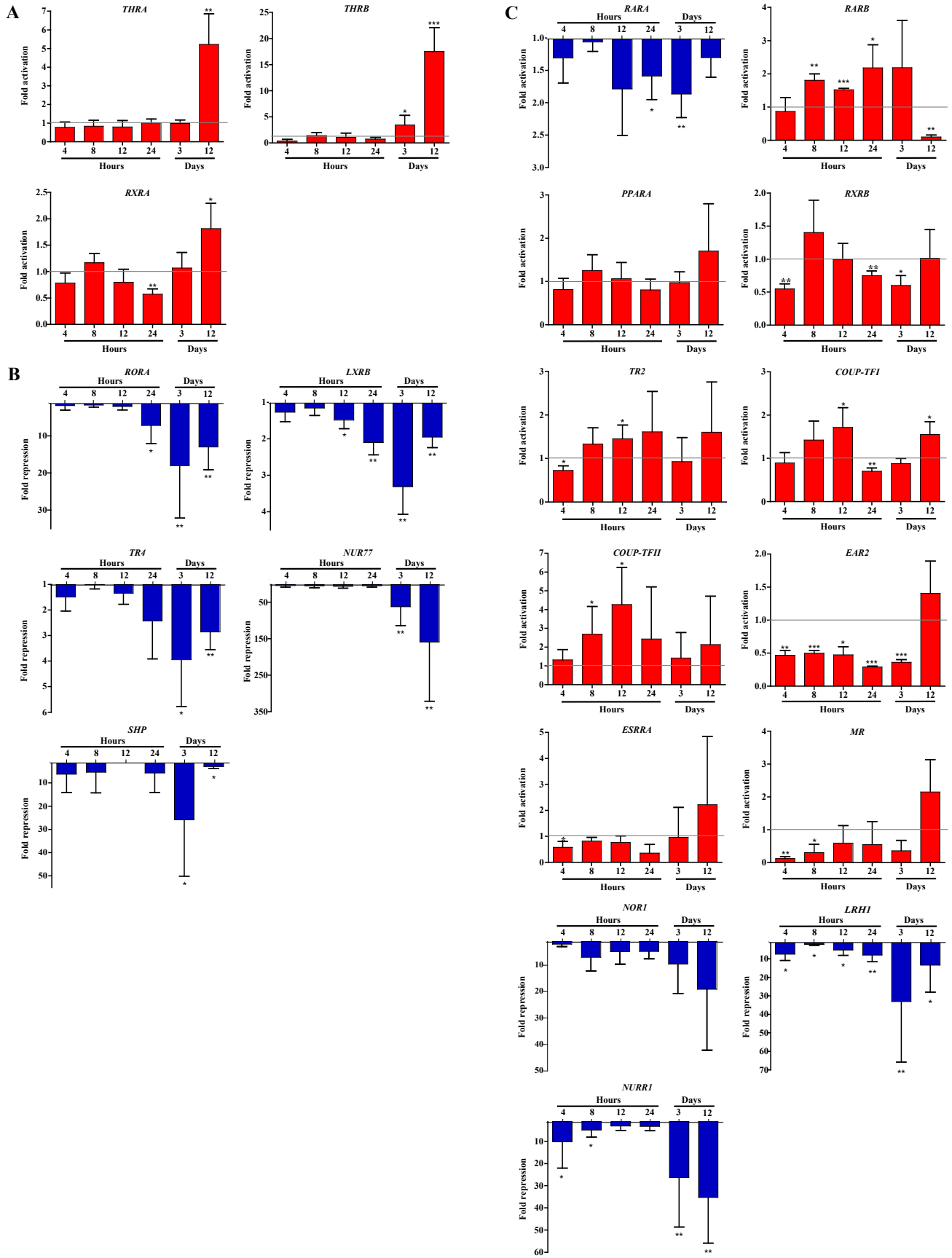

Fig. S1

Supplement: Figure S1 — Nuclear receptor expression profiling during human SGBS cell differentiation. Real-time quantitative PCR was performed in order to determine mRNA expression of nuclear receptor genes in relation to the housekeeping gene RPL13A at indicated time points of SGBS differentiation. The 21 of the 30 in SGBS cells expressed nuclear receptor genes that are not shown in Fig. 2 are displayed. Three genes were late activated (A), five were late repressed (B) and 13 showed a mixed or a modest response (C). Columns represent the means of at least three biological repeats and the bars indicate standard deviations. Two-tailed paired Student's t-tests were performed to determine the significance of the mRNA level changes in reference to undifferentiated pre-adipocytes (* p<0.05, ** p<0.01, *** p<0.001). (0.08 MB PDF) [file pone.0012991.s004.pdf]

## Quantitative PCR

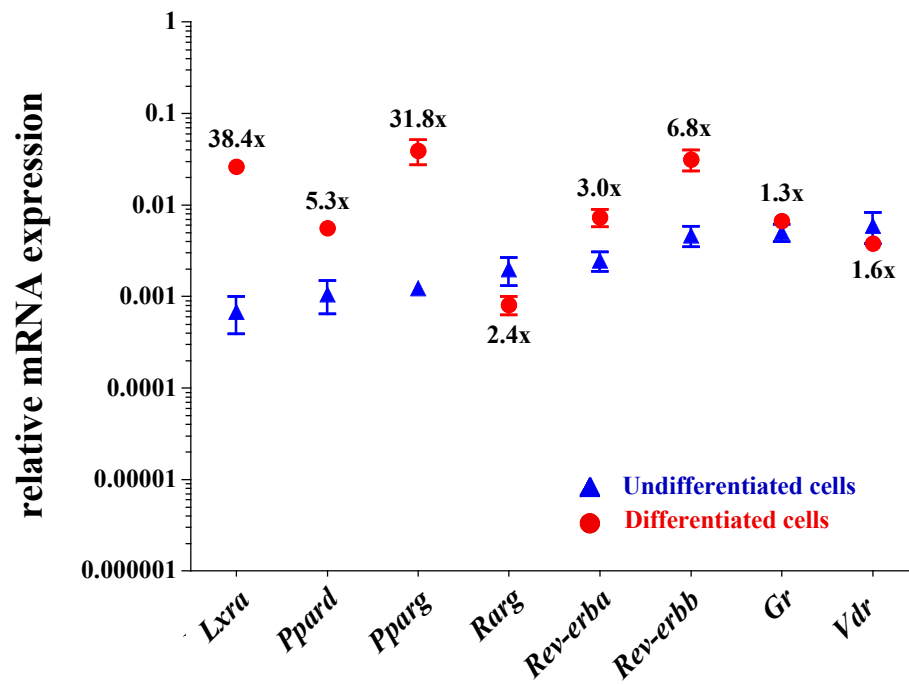

**Fig. S2**

Supplement: Figure S2 — Nuclear receptor mRNA expression in undifferentiated and differentiated mouse 3T3-L1 cells. Real-time quantitative PCR with gene-specific primers was used to determine the mRNA expression levels of eight selected nuclear receptor genes in relation to the housekeeping gene Rplp0 in undifferentiated (blue triangles) or 6 days differentiated (red circles) 3T3-L1 cells (Ar is not expressed). Fold changes were calculated in reference to undifferentiated cells. Data points represent the means of three biological repeats and the bars indicate standard deviations. (0.12 MB PDF) [file pone.0012991.s005.pdf]

# Quantitative PCR

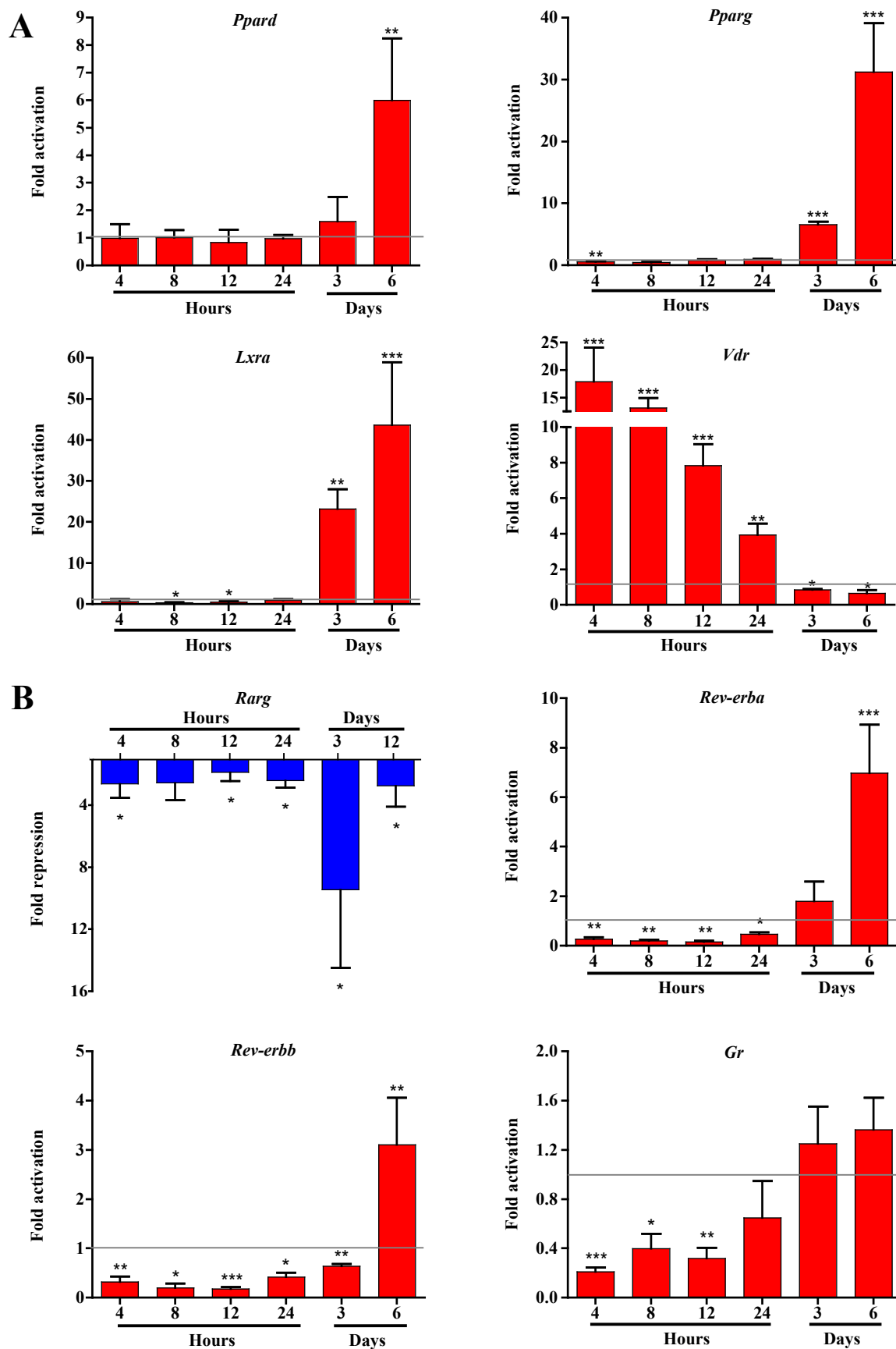

**Fig. S3**

Supplement: Figure S3 — Nuclear receptor expression profiling during mouse 3T3-L1 cell differentiation. Real-time quantitative PCR was performed in order to determine mRNA expression of eight selected nuclear receptor genes in relation to the housekeeping gene Rplp0 at indicated time points of 3T3-L1 differentiation. Four genes were mainly activated (A) and four genes were mainly repressed (B). Columns represent the means of at least three biological repeats and the bars indicate standard deviations. Two-tailed paired Student's t-tests were performed to determine the significance of the mRNA level changes in reference to undifferentiated pre-adipocytes (* p<0.05, ** p<0.01, *** p<0.001). (0.05 MB PDF) [file pone.0012991.s006.pdf]

## Public microarray data

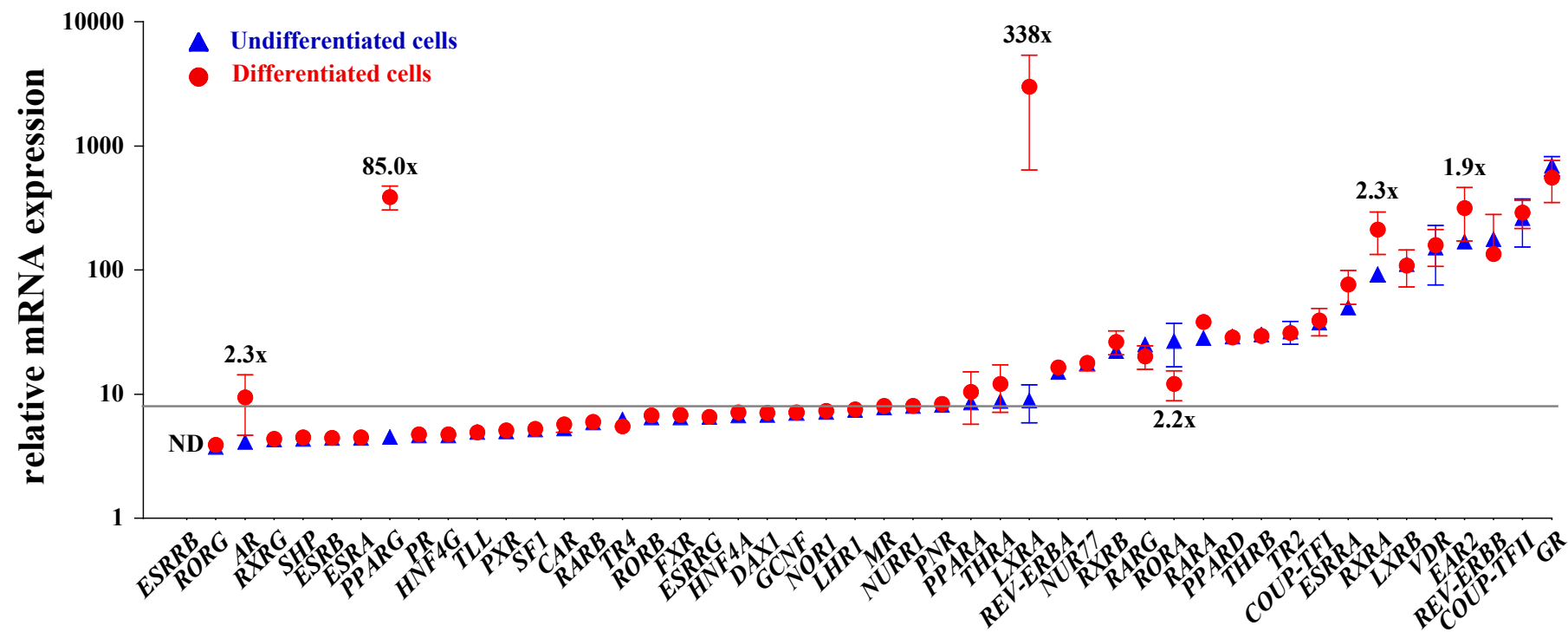

Fig. S4

Supplement: Figure S4 — Nuclear receptor mRNA expression in primary human pre-adipocytes and adipocytes. Raw data for primary human pre-adipocytes (blue triangles) or 30 days differentiated (red circles) adipocytes were obtained from the GSE1657 dataset of the public microarray repository NCBI Gene Expression Omnibus (www.ncbi.nlm.nih.gov/geo). The probe values were background corrected using the gc-rma full model, normalized using quantile normalization and summarized to gene expression values using median polish. The mRNA expression levels of all 48 nuclear receptor genes were compared and fold changes were calculated. Data points represent the means of three biological repeats and the bars indicate standard deviations. For the gene ESRRB the Affymetrix HG-U133A array did not contain any specific probe (see University of Michigan BrainArray project, http://brainarray.mbni.med.umich.edu/brainarray/default.asp). The horizontal line indicates the estimated threshold of specific expression. (0.63 MB PDF) [file pone.0012991.s007.pdf]

## Quantitative PCR

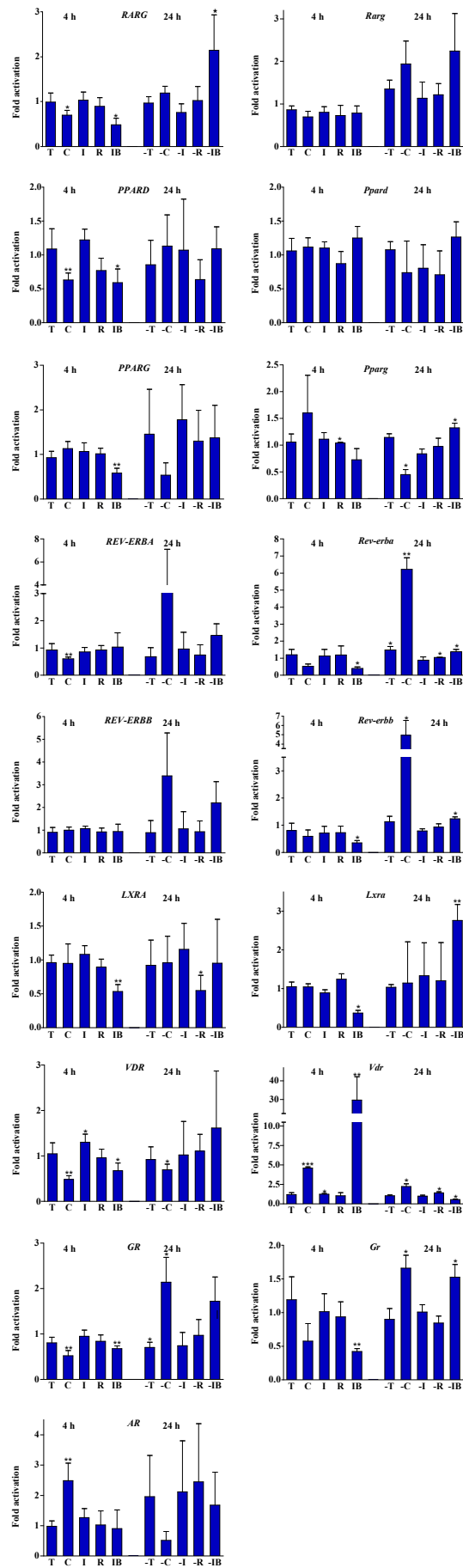

Fig.S5

Supplement: Figure S5 — Effects of individual differentiation medium compounds on nuclear receptor gene expression during early human SGBS and mouse 3T3-L1 cell differentiation. Real-time quantitative PCR was performed in order to determine mRNA expression of nine selected nuclear receptor genes in relation to the housekeeping genes RPL13A and Rplp0 in SGBS and 3T3-L1 cells, respectively. The cells were either stimulated for 4 h with either T3 (T), cortisol (C), insulin (I), rosiglitazone (R) or IBMX (IB) or they were differentiated for 24 h with full differentiation medium lacking the indicated compounds. Columns represent the means of at least three biological repeats and the bars indicate standard deviations. Two-tailed paired Student's t-tests were performed to determine the significance of the mRNA level changes in reference to undifferentiated pre-adipocytes (for 4 h treatments) or in reference to full differentiation medium (for 24 h treatments) (* p<0.05, ** p<0.01, *** p<0.001). (0.08 MB PDF) [file pone.0012991.s008.pdf]

## Red Oil staining

undifferentiated cells

differentiated cells

SGBS cells

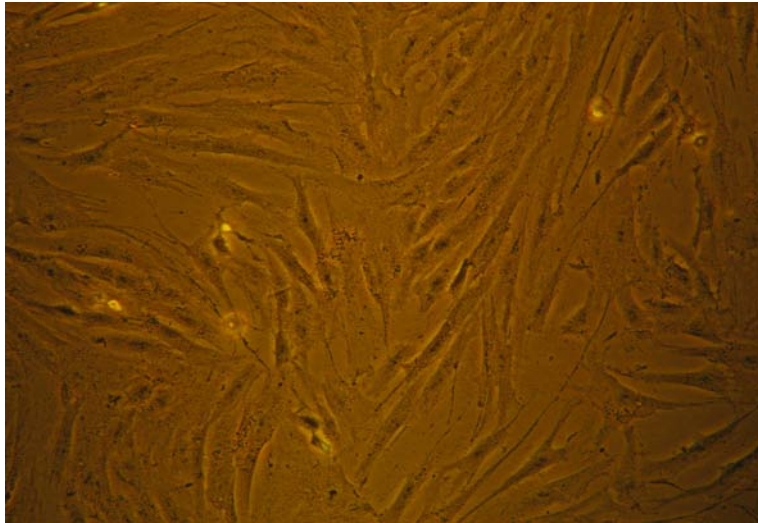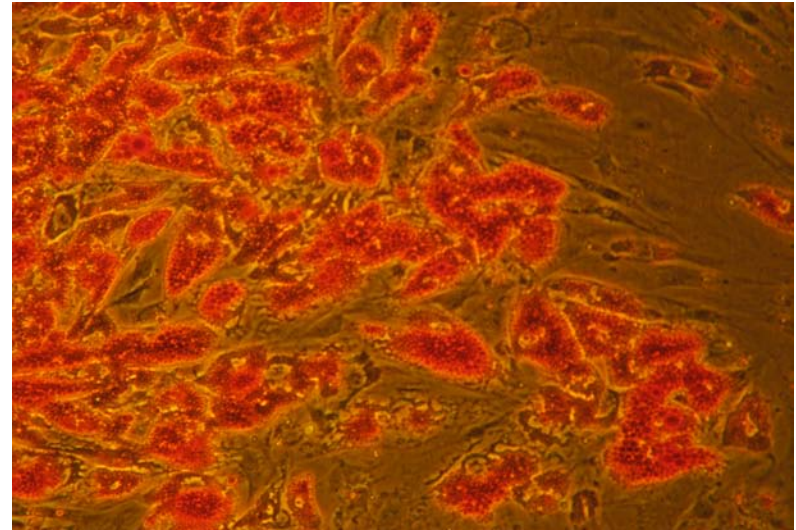

3T3-L1 cells

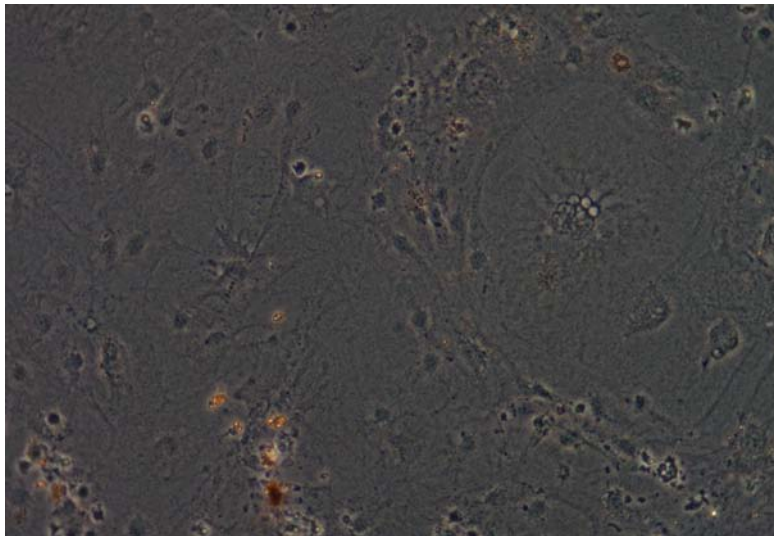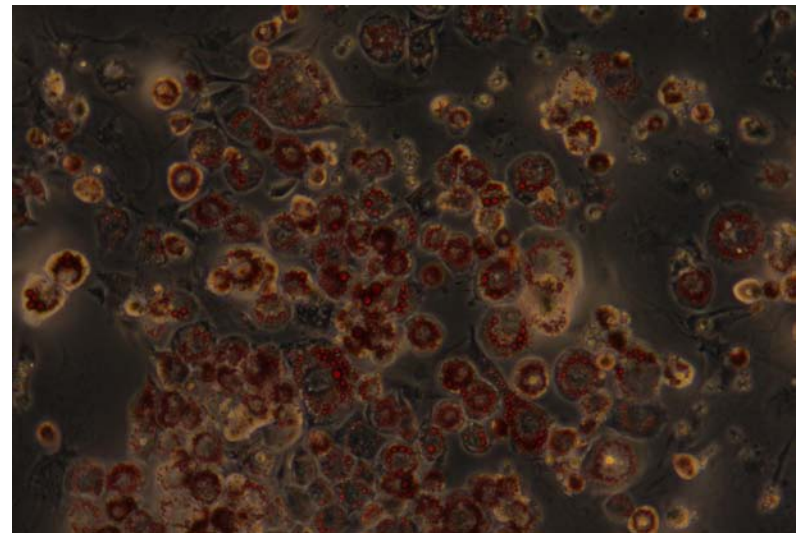

Fig. S7

Supplement: Figure S7 — Human and mouse pre-adipocytes and adipocytes. SGBS human pre-adipocytes (top left) were differentiated to adipocytes (top right) within 11 days and 3T3-L1 mouse pre-adipocytes (bottom left) were differentiated within 6 days (bottom right). Accumulation of lipid droplets was visualized by Oil Red O staining. (0.17 MB PDF) [file pone.0012991.s010.pdf]
