# Supplementary material for: Time-Resolved Expression Profiling of the Nuclear Receptor Superfamily in Human Adipogenesis
Source: PLoS One. 2010 Sep 27;5(9):e12991. doi: 10.1371/journal.pone.0012991 (PMC2946337; doi:10.1371/journal.pone.0012991)
Supplement: Figure S6 — Genomic structure of nuclear receptor genes. The UCSC genome browser was used to display the genomic regions +/− 100 kB to the TSS of nine nuclear receptor genes and the reference gene TSC22D3. GR association data to these regions were obtained from Reddy et al. (3.14 MB PDF) [file pone.0012991.s009.pdf]

Genomic locations of genes studied and public ChIP-Seq data

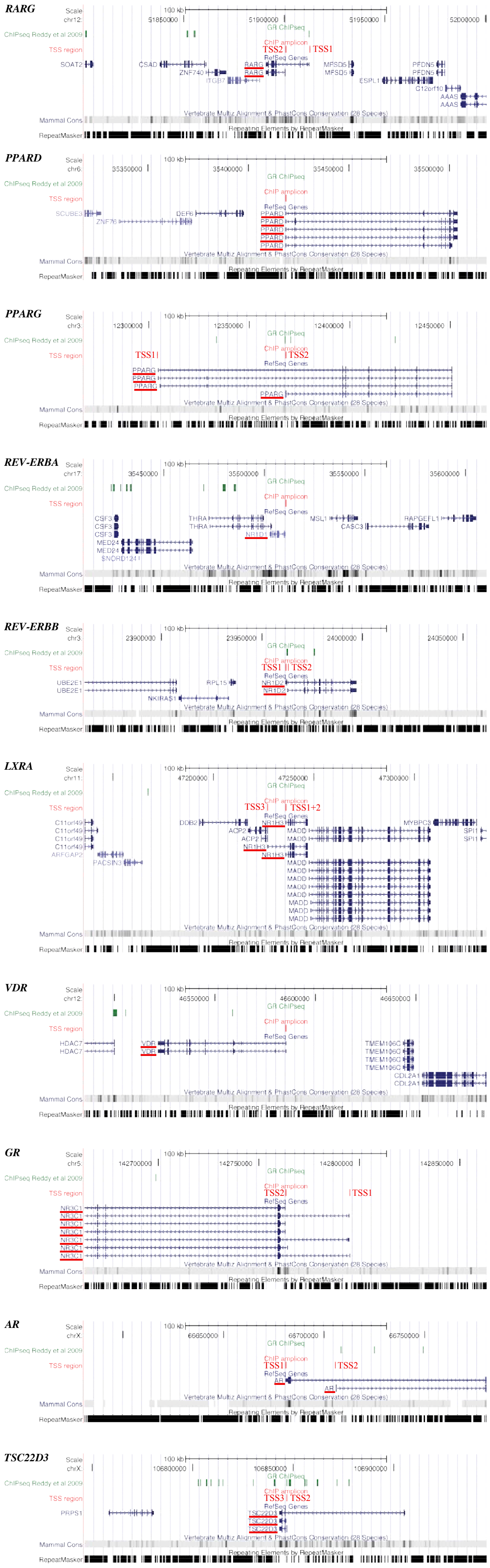

Fig. S6
